# Meta-analysis of medical cannabis outcomes and associations with cancer

**DOI:** 10.3389/fonc.2025.1490621

**Published:** 2025-04-15

**Authors:** Ryan D. Castle, James Marzolf, Miranda Morris, William C. Bushell

**Affiliations:** ^1^ Whole Health Oncology Institute, Ewa Beach, HI, United States; ^2^ Chopra Foundation, New York, NY, United States

**Keywords:** cannabis, medical Marijuana, cancer, meta-analysis, data analytics, whole health, oncology, outcomes

## Abstract

**Background:**

Growing bodies of evidence suggest that cannabis may play a significant role in both oncological palliative care and as a direct anticarcinogenic agent, but classification as a Schedule I substance has complicated research into its therapeutic potential, leaving the state of research scattered and heterogeneous. This meta-analysis was conducted to determine the scientific consensus on medical cannabis’ viability in cancer treatment.

**Objective:**

The aim of this meta-analysis was to systematically assess the existing literature on medical cannabis, focusing on its therapeutic potential, safety profiles, and role in cancer treatment.

**Methods:**

This study synthesized data from over 10,000 peer-reviewed research papers, encompassing 39,767 data points related to cannabis and various health outcomes. Using sentiment analysis, the study identified correlations between cannabis use and supported, not supported, and unclear sentiments across multiple categories, including cancer dynamics, health metrics, and cancer treatments. A sensitivity analysis was conducted to validate the reliability of the findings.

**Results:**

The meta-analysis revealed a significant consensus supporting the use of medical cannabis in the categories of health metrics, cancer treatments, and cancer dynamics. The aggregated correlation strength of cannabis across all cancer topics indicates that support for medical cannabis is 31.38× stronger than opposition to it. The analysis highlighted the anti-inflammatory potential of cannabis, its use in managing cancer-related symptoms such as pain, nausea, and appetite loss, and explored the consensus on its use as an anticarcinogenic agent.

**Discussion:**

The findings indicate a strong and growing consensus within the scientific community regarding the therapeutic benefits of cannabis, particularly in the context of cancer. The consistent correlation strengths for cannabis as both a palliative adjunct and a potential anticarcinogenic agent redefine the consensus around cannabis as a medical intervention.

**Conclusion:**

The consistency of positive sentiments across a wide range of studies suggests that cannabis should be re-evaluated within the medical community as a treatment option. The findings have implications for public health research, clinical practice, and discussions surrounding the legal status of medical cannabis. These results suggest a need for further research to explore the full therapeutic potential of cannabis and address knowledge gaps.

## Introduction

The investigation of cannabis as a potential cancer treatment has gained considerable momentum within the scientific and medical communities, driven by the promise of its therapeutic benefits in both oncological palliative care and as a direct antitumorigenic agent. This growing interest has led to a substantial body of research, offering varied insights into the efficacy, safety, and application of cannabis in oncology. Despite the abundance of studies available, there remains a distinct opacity about the level of consensus regarding the role of cannabis in cancer treatment. This discordance among researchers and clinicians is primarily attributable to several challenges inherent in the study and interpretation of cannabis research.

One of the main obstacles to reaching a unified understanding of cannabis’ effects in cancer treatment is the broad and diverse nature of the studies conducted. The existing literature spans a range of research designs, including randomized controlled trials, observational studies, and case reports, each focusing on different aspects of cannabis use in oncology, such as palliative care for chemotherapy-induced side effects or its potential as an anticancer agent. This diversity, while indicative of the wide interest and applicability of cannabis in cancer treatment, introduces significant heterogeneity into the evidence base. Variations in study design, cannabis formulations, dosages, and patient populations complicate the synthesis of data, making it challenging to draw cohesive conclusions about cannabis’ efficacy in cancer therapy.

The complexity of cannabis as a therapeutic substance further complicates research outcomes. Cannabis contains numerous cannabinoids, with tetrahydrocannabinol (THC) and cannabidiol (CBD) being the most extensively studied. These cannabinoids interact with the body’s endocannabinoid system in diverse ways, potentially leading to varied therapeutic effects, particularly in the context of cancer. Furthermore, the concentrations of these cannabinoids can differ significantly across cannabis strains and products, adding another layer of variability to research findings. The use of different cannabis formulations, ranging from synthetic cannabinoids to whole-plant extracts, further complicates comparisons between studies due to differences in cannabinoid profiles and concentrations.

Existing systematic reviews and meta-analyses on the use of cannabis in cancer care have also encountered limitations that hinder their ability to provide clear guidance. While these reviews are essential for summarizing and evaluating the evidence, many have been constrained by narrow research questions or inclusion criteria, focusing on specific cancer-related outcomes such as pain management or antitumorigenic effects. This selective approach, though necessary for maintaining a manageable scope, results in a fragmented understanding of cannabis’ full potential in oncology. There is an urgent need for more comprehensive systematic reviews that encompass a wider range of studies and employ sophisticated meta-analytic techniques to better synthesize the available data on cannabis as a cancer treatment.

To achieve a more cohesive understanding of cannabis as a cancer treatment, researchers and clinicians must address these challenges with rigorous methodology and a commitment to comprehensive analysis. Only through such efforts can the medical community fully explore and validate the therapeutic potential of cannabis in oncology, supported by a robust and unified body of evidence.

## Background

The utilization of cannabis for medicinal purposes has a storied history, extending back to ancient civilizations. The earliest recorded mention of cannabis’ medicinal application dates to 2737 BC, when it was documented in the pharmacopeia of Chinese Emperor Shen Nung (1). This ancient text described cannabis as a treatment for a multitude of ailments, including rheumatism, gout, and malaria. Subsequent historical records reveal that the medicinal use of cannabis spread across continents, finding a place in the traditional medicine practices of India, Egypt, and, eventually, Europe and the Americas.

Research into the medicinal properties of cannabis began to take a more scientific approach in the 19th century. In 1839, Irish physician William Brooke O’Shaughnessy, who had observed its use in India, introduced cannabis to Western medicine (2). His work highlighted its potential analgesic, antispasmodic, and anticonvulsant properties, laying the groundwork for future research. However, the turning point in cannabis research came in the 20th century with the isolation of THC in 1964 by scientists Raphael Mechoulam and Yechiel Gaoni (3). This discovery, identifying the psychoactive compound responsible for cannabis’ effects, propelled further scientific inquiry and helped elucidate the plant’s pharmacological properties.

Despite these advancements, the legal classification of cannabis as an illicit substance in many parts of the world, notably marked by the United States’ Controlled Substances Act of 1970, has posed significant challenges for researchers. Cannabis’s designation as a Schedule I substance, deemed to have a high potential for abuse and no accepted medical use, has restricted access for research purposes. This legal status has contributed to inconsistencies and heterogeneity in cannabis research in several ways:

1. **Limited Research Funding**: The classification has often deterred funding agencies from allocating resources to cannabis research, leading to a scarcity of high-quality studies, particularly those involving clinical trials (4).
2. **Restricted Access to Cannabis**: Researchers have faced challenges in obtaining cannabis of consistent quality and composition for study, complicating efforts to produce the standardized, replicable research necessary to determine efficacy and safety profiles (5).
3. **Varied Legal Status across Regions**: The differing legal status of cannabis across states and countries has resulted in a fragmented research landscape, where methodologies and research priorities vary widely, further contributing to the heterogeneity of findings (6).
4. **Reliance on Self-Reported Data**: Owing to difficulties in conducting controlled experiments, researchers have often had to rely on observational studies or self-reported data, which may introduce biases or inaccuracies (7).

These obstacles have not only slowed the pace of cannabis research but also led to a body of literature characterized by a wide range of study designs, participant populations, cannabis formulations, and outcome measures. The result is a complex and often contradictory evidence base that reflects the legal and logistical hurdles faced by the scientific community in exploring cannabis’ medicinal potential.

### Oncological palliative care

The most frequent use of medical cannabis in relation to cancer has been as an adjunctive palliative treatment for the side effects of traditional cytotoxic antineoplastic agents, such as chemotherapy (8). The analgesic properties of medical cannabis have been extensively studied, with cannabinoids showing efficacy in modulating pain through interactions with the endocannabinoid system. Cannabinoids like THC and CBD engage with cannabinoid receptors (CB1 and CB2) in the central and peripheral nervous systems, altering pain perception pathways and providing relief from chronic pain, including neuropathic pain (9). Clinical trials have demonstrated that cannabis and its derivatives can significantly reduce patient-reported pain scores with minimal risks (10).

Cannabis has historically been widely used to mitigate chemotherapy-induced nausea and vomiting (CINV) due to its active compounds, primarily THC and CBD. These cannabinoids interact with the body’s endocannabinoid system, particularly the CB1 receptors in the central nervous system, which play a role in regulating nausea and vomiting. By activating these receptors, cannabis can effectively reduce the severity of CINV, a common and debilitating side effect of chemotherapy. Clinical studies and anecdotal evidence have consistently supported the efficacy of cannabis in this context, leading to its recognition as a therapeutic option for patients undergoing cancer treatment (11).

Cannabis has been used to address appetite problems in chemotherapy patients, primarily due to its ability to interact with the endocannabinoid system, which plays a significant role in regulating hunger and metabolism. However, the results reported in studies have been heterogeneous and though many studies have shown cannabis to be effective in increasing appetite and countering weight loss in chemotherapy patients, some have reported minimal effects or inconsistent outcomes (12). Despite this variability, cannabis remains a widely considered option for managing appetite issues in chemotherapy, supported by both clinical use and patient-reported outcomes.

### Efficacy as a treatment

The exploration of cannabis in cancer treatment has focused on symptom management, including pain, nausea, and cachexia, with a relatively recent focus on the potential direct antitumor effects of cannabinoids. Patient-reported outcome measures indicate that 70%–90% of patients who used cannabis to directly treat cancer symptoms reported improvements, with less than 5% reporting adverse effects (13). Preclinical studies have shown that cannabinoids can induce apoptosis and inhibit tumor growth in various cancer cell lines (14). Clinical evidence supports the efficacy of cannabinoids in managing chemotherapy-induced nausea and improving appetite in cancer patients (15). The investigation into cannabinoids’ direct anticancer properties is ongoing, with emerging data suggesting a promising yet complex therapeutic potential that requires further elucidation through rigorous clinical research.

### Select literature review

A significant body of research, including numerous studies, systematic reviews, and meta-analyses, has been dedicated to exploring the medical viability of cannabis. However, the development of evidence-driven standards of care has been hindered by the lack of a clear consensus within the literature. This challenge is compounded by the heterogeneity of study designs, patient populations, and cannabis formulations, which often result in contradictory findings. The divergent outcomes reported across these studies are seen in **Table 1** and reflect the complexity of cannabis as a therapeutic agent as well as the need for more expansive research methodologies.

- De Feo et al. (2023) (16)
- To et al. (2023) (17)
- Zeraatkar et al. (2022) (18)
- Lee et al. (2023) (19)
- Hanganu et al. (2022) (20)
- Valenti et al. (2022) (21)
- Bachari et al. (2020) (22)

**Table 1:** Select overview of existing reviews into medical cannabis.

Study	Purpose	Methods	Limitations	Cannabis Benefits Reported	Benefit Described	Cannabis Adverse Effects	Adverse Effects Described	Cannabis Use Supported
De Feo G, et al. (2023) (16)	Develop guidelines for cannabis as psychological treatment for cancer patients	Literature review comparing placebo vs. active alternative	Few included studies assessed the efficacy of cannabis	Yes	Sleep improvement, mood improvement	No	None	*No*
To J, et al. (2023) (17)	Develop guidelines and track adverse effects for cannabis as pain treatment for cancer patients	Systematic review of RCT’s comparing placebo vs. active alternative	Inconsistency measuring the types and levels of harm	Yes	Reduced cancer-related pain	Yes	Potential risk of harm too dangerous for cancer patients	*No*
Zeraatkar D, et al. (2022) (18)	To establish the prevalence of serious harms of cannabis used for pain management	Systematic review and meta-analysis on adverse effects	Evidence overall was of low confidence	Yes	Pain management with fewer adverse effects than opioids	Yes	Approximately 13% of patients had adverse effect, generally paranoia or nervousness	*Yes*
Lee C, et al. (2023) (19)	To determine the efficacy of cannabis for lower back pain (LBP) in the context of pain levels and opioid use	Literature review focusing on adults with LBP taking medical cannabis and with option of opioids	Mostly observational rather than RCT studies	Yes	Pain management equivalent to opioids, decreased opioid dependence	No	None	*Yes*
Hanganu B, et al. (2022) (20)	Synthesize evidence of cannabis’ efficacy for cancer patients	PRISMA guidelines	Insufficient RCTs	Yes	Popular adjunctive treatment for glioblastoma	No	None	*Inconclusive*
Valenti C, et al. (2022) (21)	Examine the biological effects of CBD on various human pathological and cancer cell populations	PRISMA guidelines involving human cell lines and cultures from non-healthy donors, with CBD	Study was limited to *in vitro* research	Yes	Reduced proliferation and metastasis of cancer cells, increased apoptosis and anti-inflammatory regulation		None	*Yes*
Bachari A, et al. (2020) (22)	Review the existing *in vivo* evidence on the effects of cannabinoids as direct treatment of melanoma	Systematic review to identify *in vivo* studies on the effects of cannabinoids on melanoma	Small number of eligible *in vivo* studies	Yes	Increased apoptosis of cancer cells, antitumorigenic effects	No	None	*Yes*

The full analysis of each review along with the following synopsis is included in **Appendix A**.

Taken in aggregate, the seven systematic reviews and meta-analyses discussed in this literature review, which collectively encompass reviews of over a thousand studies, illustrate the inconsistent and often contradictory nature of current research on medical cannabis. These reviews present conflicting conclusions on key topics, including the presence and significance of health metrics, the efficacy of cannabis as an adjunct for managing cancer treatment symptoms, the overall viability of cannabis for cancer patients, and the nature and prevalence of adverse effects. The inconsistency in findings across these studies highlights the challenges in drawing definitive conclusions from the existing body of research. Addressing these issues requires more than isolated systematic reviews; it necessitates a comprehensive and inclusive meta-analysis approach that leverages big data to provide a more robust and reliable assessment of cannabis’ medical potential.

### Diversity of cannabinoids and cancers

The therapeutic potential of medical cannabis in oncology is a rapidly evolving field, yet it is complicated by the broad heterogeneity of both cannabis extracts and cancer subtypes. This complexity highlights the necessity of a large-scale analytical summary when evaluating cannabinoid-based interventions for cancer treatment.

Cannabis is a chemically complex plant, with over 100 cannabinoids and numerous terpenes contributing to its pharmacological effects. The two most extensively studied cannabinoids, THC and CBD, exert different, and sometimes complementary, effects on cancer biology. However, cannabis-based formulations are not limited to THC and CBD alone. Full-spectrum extracts contain a broader range of bioactive compounds, including cannabigerol (CBG), cannabichromene (CBC), flavonoids, and terpenes, all of which contribute to what is known as the entourage effect (23). This phenomenon suggests that cannabinoids and terpenes may work synergistically to enhance therapeutic efficacy, modulate bioavailability, and mitigate adverse effects. Despite this complexity, many studies have focused primarily on CBD, largely due to regulatory restrictions on THC and the need to avoid its psychoactive effects (24). While CBD demonstrates anti-inflammatory, pro-apoptotic, and antiproliferative properties in preclinical cancer models, its effects in isolation do not mirror those of full-extract cannabis, which includes THC and other bioactive compounds, suggesting that a reductionist approach to cannabinoid-based therapy may not be appropriate.

Just as cannabis extracts vary widely in their composition, cancer itself is not a monolithic disease but a collection of highly heterogeneous malignancies. Each cancer type is characterized by unique genetic, molecular, and histopathological features that influence its progression and treatment response. Research has demonstrated that cannabinoid efficacy differs by both breast cancer subtype and cannabinoid composition, with some forms of breast cancer responding more favorably to different cannabinoid treatments than others (25).

Beyond breast cancer, other cancers exhibit diverse responses to cannabinoids. Colorectal, liver, pancreatic, skin, prostate, and glioblastoma cancers have been studied for their susceptibility to cannabinoid-induced apoptosis, autophagy, and cell cycle arrest. The results have varied widely by cannabinoids and/or cancer type, with some differential effects possibly based on receptor expression profiles (26). The variable cannabinoid receptors (CB1, CB2, and GPR55) in cancer progression further complicates an already complex landscape, as receptor expression varies between both individuals and cannabinoids.

Given the substantial heterogeneity in both cannabis extracts and cancer subtypes, a large-scale perspective is necessary to identify patterns. It is overly simplistic to treat medical cannabis as a therapeutic agent that can be reduced to a single block of interactions, and determining efficacy on single studies is likely to result in unreliable results. System-based strategies could identify cannabinoid-responsive cancers through large-scale evidence-driven approaches, developing targeted cannabinoid formulations that consider the ratio of THC, CBD, and minor cannabinoids, and investigating interactions with standard-of-care treatments such as chemotherapy, immunotherapy, and targeted therapies. Future clinical trials should move beyond generic CBD-based studies to explore the full therapeutic potential of comprehensive cannabinoid formulations, incorporating THC and other cannabis-derived compounds where legally and ethically feasible.

Further discussion of the relevance of including the full range of cannabinoids is listed in **Appendix A**.

### Sentiment analysis and public health policy

Sentiment analysis, a subfield of natural language processing (NLP) and machine learning, has emerged as a powerful tool in big data analytics, offering a systematic method to assess subjective information and emotional undertones embedded within vast amounts of text. At its core, sentiment analysis attempts to classify expressions of sentiment—typically categorized as positive, neutral, or negative—by applying computational techniques to unstructured textual data. By converting qualitative language into structured, quantifiable data, sentiment analysis enables researchers to analyze trends, extract insights, and identify patterns that would otherwise be difficult to discern manually (27). In the context of a large-scale meta-analysis, particularly one encompassing thousands of studies on medical cannabis, sentiment analysis serves as a practical tool for synthesizing extensive and diverse findings across multiple disciplines and research methodologies.

One of the primary advantages of sentiment analysis is its ability to process and analyze massive datasets far beyond the capacity of human reviewers. Traditional systematic reviews and meta-analyses require extensive manual effort, often taking months or years to complete, whereas sentiment analysis can expedite this process by rapidly extracting patterns from text-based research. It allows for the identification of overarching themes, research trends, and shifts in scientific consensus over time. In medical research, sentiment analysis can highlight the prevailing attitudes toward specific treatments, uncover the strength of consensus regarding therapeutic efficacy or risks, and even provide a sense of how different areas of research evolve in response to new findings. By mapping the sentiment expressed in large collections of studies, researchers can gain a clearer understanding of how a particular intervention, such as medical cannabis, is perceived across different clinical settings, patient populations, and regulatory landscapes (28).

Despite these advantages, sentiment analysis is not without limitations, particularly in the context of scientific literature. One major challenge is the inherent complexity and nuance of medical research language, which does not always conform to the straightforward positive–negative dichotomy that sentiment analysis models often employ. Scientific discourse is frequently neutral, analytical, and highly technical, making it difficult for sentiment classification algorithms to discern evaluative meaning from purely descriptive or methodological language. Furthermore, sentiment analysis algorithms may struggle with ambiguity, indecision, or context-dependent meanings, leading to potential misinterpretations. This is particularly relevant in medical literature, where a negative sentiment in one context—such as describing the progression of a disease—does not necessarily imply a negative evaluation of a treatment or intervention.

Another key concern is that sentiment analysis often relies on pre-existing lexicons or supervised learning models trained on general language corpora, which may not be well-suited for specialized medical terminology. The context in which terms appear is crucial, as a single word may carry different connotations depending on its surrounding text. Additionally, scientific papers frequently employ cautious or tentative language, such as “further research is needed” or “preliminary evidence suggests,” which may be misclassified as neutral or negative sentiment despite indicating promising yet inconclusive findings. This underscores the importance of refining sentiment analysis models for medical applications by incorporating domain-specific training data and contextual language processing.

To mitigate these challenges, researchers utilizing sentiment analysis in medical meta-analyses must adopt rigorous validation methods, including cross-referencing algorithmic findings with expert human evaluation and employing hybrid models that combine rule-based and machine learning approaches. Additionally, researchers must be transparent about the limitations of sentiment analysis and interpret findings within the broader context of the literature rather than treating sentiment scores as definitive indicators of scientific consensus.

## Methodology

### Definitions

It is necessary to define key terms to ensure clarity and precision in understanding the research approaches and findings. Definition of terms and methods are listed in **Appendix A**.

### Search terms

Tracking the development of search terms is essential for establishing a reliable literature base. This task involves precision to ensure the inclusion of studies pertinent to the research objectives. For a database such as PubMed, which contains a wide array of biomedical literature, well-defined search terms help in retrieving studies that are most relevant to the topic of medical cannabis. This categorization aids in organizing the studies for more detailed analysis, allowing for a systematic approach to handle the volume of data. The 47 search terms often combine specific keywords and Medical Subject Headings (MeSH) to refine the search process, aligning it closely with the research question. Compiling search terms and initial results totaled 59,071 initial studies.

The breadth of search terms includes terms directly and indirectly related to cancer and cannabis, in order to capture tangential findings as well as provide data for further analysis. The full listing of all search terms, keywords, and categorizations is provided in **Appendix A**, and the listing of search terms is found in **Appendix A**, **Table 1**. The subsequent phase of the review involves filtering out studies that do not directly contribute to the research question or are duplicates. This step is necessary for refining the pool of studies by removing those that lack text to be analyzed, include contents that cannot be interpreted with available technology, or represent repeated entries of the same study. The aim is to ensure that each study included in the review is unique and directly relevant to the topic of medical cannabis.

**Figure 1:**
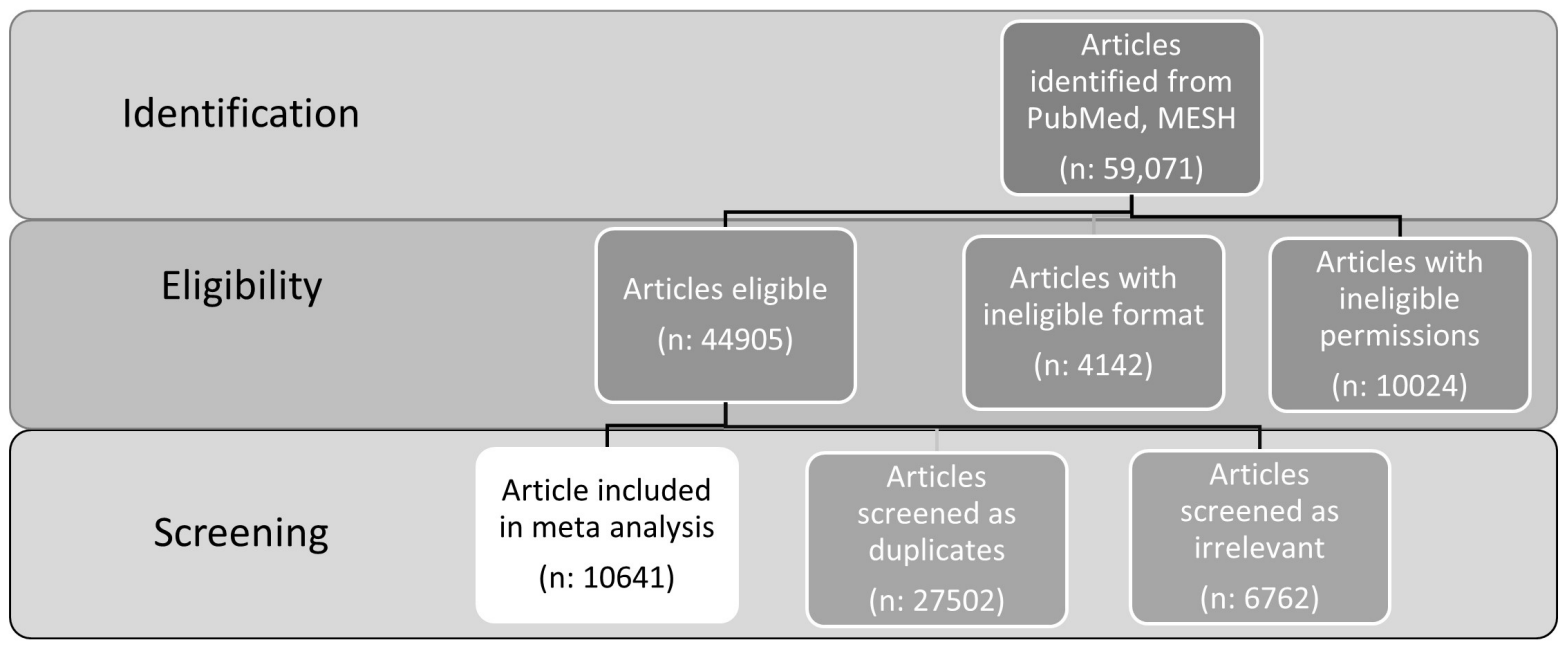
Inclusion process for meta-analysis. Initial body of articles identified through PubMed and MeSH, followed by eligibility filter by formatting and copyright accessible permissions, resulting in a final screening that eliminated duplicates and irrelevant articles, resulting in 10641 relevant, unique studies.

Below in **Figure 1** is the PRISMA style flow diagram of the inclusion process of articles.

After this filtering process, the number of studies that remain and are deemed relevant for the review and analysis is 10,641. This figure represents the collection of literature that will be subjected to further analysis, such as quality assessment and data extraction, as part of the systematic review process. This approach ensures that the review is based on a comprehensive and relevant set of studies, facilitating a robust analysis of the available evidence on medical cannabis.

A full explanation of the selection process is provided in **Appendix A**. Achieving parity in the allocation of keywords for sentiment analysis is vital to ensure unbiased interpretation of evidence. With 97 keywords identified for supporting evidence and 102 keywords for not supporting evidence, the distribution is relatively balanced, with supporting keywords constituting approximately 48.7% and not supporting keywords approximately 51.3% of the total keywords allocated for these sentiments (199 keywords in total). This close percentage distribution ensures that the sentiment analysis does not inherently favor one viewpoint over the other due to an imbalance in keyword quantity. The allocation for keywords suggesting unclear evidence stands at 55, which is significantly lower compared to the other two categories. The proportion of unclear keywords is approximately 21.6% when considering the total count of keywords across all three sentiment categories (254 keywords in total).

### Correlations

Correlations, also referred to as associations, are employed as the primary metric to elucidate patterns and associations within the extensive dataset of medical cannabis research. Correlation coefficients, such as Pearson’s *r*, are statistical measures that quantify the degree to which two variables are related. While correlation does not explain causation, correlation patterns can nonetheless help predict outcomes. Full discussion of methods, calculations, and association strengths is provided in **Appendix A**.

#### Keyword occurrences

The methodology for tracking the occurrence of keywords within individual studies in the systematic review dataset involves a detailed, quantitative approach to textual analysis. This method is instrumental in uncovering patterns and themes across a large volume of literature on medical cannabis, facilitating a nuanced understanding of the research landscape, and is detailed in **Appendix A**.

#### Dominant instances/sensitivity analysis

The potential for keyword occurrences to skew findings is a notable concern, particularly when outlier occurrence rates or the inclusion of banal terms may disproportionately influence the sentiment portrayal. Outlier occurrence rates can unduly affect the sentiment analysis by overemphasizing sentiments that are not representative of the overall narrative of each article, while banal terms, which are commonly used but carry minimal sentiment weight, could act as confounders, diluting the strength and clarity of sentiment signals within the data. An alternative analytic lens could provide additional insights or clarifications.

To address these issues, a separate methodological approach was employed, recalculating sentiment analysis through the lens of sentiment-related keyword preponderance and assigning a zero-sum sentiment dominance to each study. This method aims to neutralize the impact of outliers and mitigate the influence of banal term confounders, providing a more balanced and accurate reflection of the sentiment landscape. By doing so, studies are weighted based on the dominance of sentiment-expressive keywords, thereby ensuring that the sentiment portrayal is more reflective of the comprehensive and nuanced views within the literature.

*Example: A study that had 10 keywords associated with a supported sentiment, 5 keywords associated with the not supported sentiment, and 7 keywords associated with the unclear sentiment would be listed as dominant in the supported sentiment.*

In addition to providing insights independently, comparing the results of sentiment dominant instances with those obtained from the initial keyword occurrence rates serves the function of sensitivity analysis. This comparative approach seeks to validate the original findings by demonstrating consistency across different analytical methodologies. If the sentiment dominant instance results show similarity to the keyword occurrence outcomes, it suggests methodological efficacy in the primary analysis, affirming that the sentiment trends identified are robust and not artifacts of analytical biases. Such an approximate similarity in results would bolster confidence in the primary analysis, confirming its capacity to accurately capture and reflect the prevailing sentiments within the extensive body of research on the topic.

Sensitivity analyses have limitations. While they can reveal the presence of potential instability in the findings, they do not necessarily pinpoint the specific sources of such variability. Additionally, the process requires assumptions about alternative measurement strategies, which may not cover all possible variations in the analysis.

### Categories

The grouping of correlations under different tiers allows for greater clarity and granularity in analyzing patterns at both a large and a small scale. To that end, individual keywords have been grouped under topics and the topics have been grouped under categories. Full descriptions and justification for these categorizations are found in **Appendix A**.

Please note that inflammation both can act as a symptom of cancer and can be a contributing factor toward cancer risk. Furthermore, not all cancers produce significant inflammation and not all inflammation increases the risk of cancer. Inflammation is included as a general health metric with the understanding that this study does not purport to define the causal relationship between inflammation and cancer.

#### Health metrics

- (Anti-) Inflammation
- Therapeutics

#### Cancer treatments

- Appetite
- Chemotherapy
- Nausea
- Opioids
- Pain
- Immune therapy

#### Cancer dynamics

- Anticarcinogenic
- Cancerous
- Cancers
- Tumor growth
- Tumor size
- Remission

### Refined data

For a correlation to be included in the final results to be analyzed, it must be considered substantive and reliable, and so must exhibit not only a measurable strength, as indicated by the correlation coefficient *r*, but also statistical significance, denoted by a *p*-value. In this analysis, each category and topic will undergo a significance assessment to determine the robustness of the observed correlations. To ensure the integrity and reliability of the findings, only those correlations with a *p*-value equal to or lower than 0.05 will be retained for the refined dataset. This threshold for significance, *p* < 0.05, adheres to standard statistical conventions, marking the boundary at which results are considered statistically significant, and thereby unlikely to have occurred by chance, as outlined in **Table 2**.

- Insignificant (excluded): *p* > 0.05.
- Acceptable significance (probable trend): *p* = 0.01 to 0.05
- High significance (very probable trend): *p* = 0.0001 to 0.009
- Very high significance (consistent trend): *p* < 0.0001

**Table 2:** p-values indicating level of significance by calculations using t-scores, used to determine inclusion in the refined final results.

*p-*value	Interpretation
*p > 0.05*	Insignificant (excluded)
*p = 0.01 to 0.05*	Acceptable significance (probable trend)
*p = 0.0001 to 0.009*	High significance (very probable trend)
*p < 0.0001*	Very high significance (reliable trend)

Significance is indicated by shades of blue, darker colors indicating a smaller p-value. Pink coloration indicates unacceptable significance.

Significance often does not, by itself, provide actionable information, a methodological oversight common in research. Therefore, any results to be analyzed must also demonstrate at least very weak strength of association (*r* = −0.01 to 0.01) in order to be considered indicative of a meaningful pattern, as indicated in **Table 3**.

**Table 3:** Correlation strengths according to modified Pearson’s r, color coded with positive to negative association strengths.

	***+1.00:*** *Perfect positive linear relationship.*
	***+0.90 to +0.99*** *: Very strong positive relationship.*
	***+0.70 to +0.89*** *: Strong positive relationship.*
	***+0.50 to +0.69*** *: Moderate positive relationship.*
	***+0.30 to +0.49*** *: Mild to moderate positive relationship.*
	***+0.05 to +0.29*** *: Weak positive relationship.*
	***+0.01 to +0.05*** *: Very weak positive relationship.*
	***−0.009 to +0.009*** *: No linear relationship.*
	***−0.01 to +0.05*** *: Very weak inverse relationship.*
	***−0.05 to −0.29*** *: Weak inverse relationship.*
	***−0.30 to −0.49*** *: Mild to moderate inverse relationship.*
	***−0.50 to −0.69*** *: Moderate inverse relationship.*
	***−0.70 to −0.89*** *: Strong inverse relationship.*
	***−0.90 to −0.99*** *: Very strong inverse relationship.*
	***−1.00*** *: Perfect inverse linear relationship.*

Inclusion in the Refined Dataset requires:

- *p* < 0.05

and

- *r* > 0.01 or *r* < −0.01

The complexity and heterogeneity inherent in medical cannabis studies make the task of identifying and measuring consensus challenging. In such a context, the combined assessment of correlation strength and statistical significance becomes particularly crucial. It allows researchers to discern subtle but consistent trends within the data, thereby providing a more nuanced and comprehensive understanding of the evidence landscape.

## Results

### Initial data

Correlations were calculated between all individual keywords within a topic and the sentiments of supported, not supported, and unclear, for both keyword occurrences and dominant instances. Given the large quantity of correlations initially examined, only sentiment analysis results will be provided here. The refined dataset subsection will list the correlations meeting significance and association strength minimums, the full list of unfiltered results can be found in **Appendix A**.

#### Sentiment analysis

**Figure 2:**
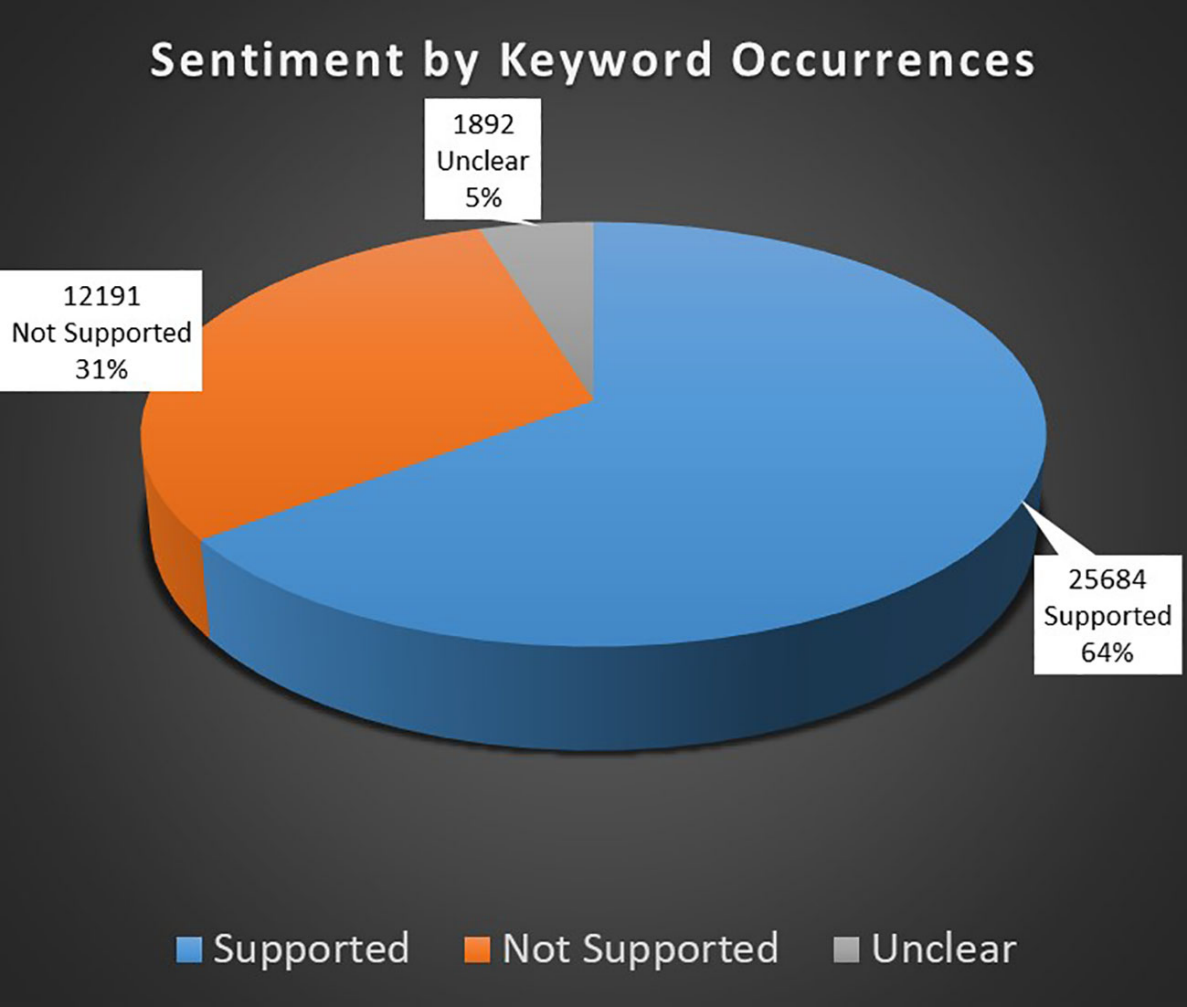
Proportion of keywords allocated to different sentiments as keyword occurrences.

The results section of the study begins with an analysis of sentiments expressed in the medical cannabis research literature. The sentiment analysis quantitatively assessed the occurrence of terms associated with three sentiment categories: support, not supported, and unclear. These findings, listed in **Table 4** and illustrated in **Figure 2**, reveal the distribution of sentiment across the studies analyzed. 

**Table 4:** List of total sum of occurrences of keywords allocated as indicative of sentiments.

Sentiment	Total Occurrences
Supported	25,684
Not Supported	12,191
Unclear	1,892

##### Keyword occurrences

There were 39,767 occurrences of keywords associated with any of the supported, not supported, or unclear sentiments. The analysis yielded a total of 25,684 unique occurrences of terms that suggest support for the efficacy or benefits of medical cannabis, constituting 64.5% of all sentiment-related occurrences. This significant majority indicates a predominant inclination in the literature toward findings that support the use of medical cannabis for various conditions or therapeutic purposes. The sentiment analysis as identified by keyword occurrence are depicted in **Figure 1**.

Terms indicating a sentiment of not being supported by the evidence amounted to 12,191 occurrences, accounting for 30.6% of the sentiment occurrences. This substantial but notably smaller fraction compared to the supported category suggests that while there is considerable research casting doubt or presenting negative outcomes associated with medical cannabis, it is less prevalent than research supporting its use.

Occurrences of terms indicating unclear or indeterminate results were the least frequent, totaling 1,892 and representing 4.7% of all sentiment occurrences. This smallest proportion is consistent with the relative rarity of studies that yield thoroughly inconclusive or ambiguous results regarding medical cannabis.

The disparity between the numbers of occurrences of supported versus not supported terms, despite an approximate equivalence in the number of search terms for both categories, is noteworthy. This discrepancy suggests a tendency within the corpus of analyzed literature to report findings favorable to medical cannabis. The Analysis section will discuss the likelihood that such a pattern could reflect broader trends in research funding, publication biases, or inherent challenges in conducting and reporting studies on medical cannabis.

##### Topic dominant instances

The sensitivity analysis was conducted by recalculating the keywords, sentiment analyses, and correlation coefficients, which were initially based on the frequency of keyword occurrences, to instead focus on zero-sum sentiment dominance—supported, not supported, and unclear—within the dataset. The sentiment dominance was determined by the ratio of keywords corresponding to each sentiment category to the total sentiment-expressive keywords in each study. This methodological shift from KO to dominant instances (DI) provided a basis to assess how changes in the analytical focus affect the established relationships between sentiments.

*Example: A study that had 10 keywords associated with a supported sentiment, 5 keywords associated with the not supported sentiment, and 7 keywords associated with the unclear sentiment would be listed as supported-dominant, the article counting as 1 dominant instance (DI), as demonstrated in* **Table 5**.

**Table 5:** Sentiment analysis comparison of dominant instances (DI) among articles vs. keyword occurrences (KO) among all articles.

Sentiment	Dominant Instances	DI %	Keyword Occurrences	KO %
Supported	5,689	71.4%	25,684	64.6%
Not Supported	2,041	25.6%	12,191	30.7%
Unclear	235	3.0%	1,892	4.8%

Complete comparison of all topics via keyword occurrence and dominant instance is available in **Appendix A**.

The calculated sentiment dominance revealed 5,689 instances of articles predominantly supporting the efficacy or benefits of medical cannabis, 2,041 instances where the sentiment was predominantly not supported, and 235 instances where the dominant sentiment was unclear. Proportionally, sentiment dominance analysis illustrated that 71.4% of the articles exhibited a predominant alignment with supported sentiments, 25.6% with not supported sentiments, and only 2.9% with unclear sentiments.

The sensitivity analysis conducted via sentiment dominance showcases a pattern consistent with the initial sentiment analysis by keyword occurrences. However, the dominance calculation reveals a more pronounced inclination toward supported sentiments, suggesting that when articles are assessed on the aggregate sentiment conveyed, the support for medical cannabis is more substantial.

This comparative analysis between sentiment analysis based on keyword occurrences and sentiment dominance supports a congruent trend, though with a discernible intensification of support in the latter. **Table 6** demonstrates that the association between keyword occurrence and dominant instance sentiments has the strongest association strengths and *p*-values seen in this analysis. This suggests that the methodological approach is sound and the assigned sentiments are reasonably reliable.

**Table 6:** Correlation between keyword occurrence and dominant instance methods of sentiment analysis.

Keywords	S KO	S KO p	NS KO	NS KO p	U KO	U KO p	S DI	S DI p	NS DI	N DI p	U DI	U DI p
**Supported KO**							0.585866514	0	-0.2820384	9E-194	-0.1232888	3E-37
**Not Supported KO**							−0.274465797	3E−183	0.5624197	0	−0.0874223	2E−19
**Unclear KO**							−0.071240068	2E−13	−0.0179591	0.064	0.369684	0
**Supported DI**	0.585866514	0	−0.2744658	3E−183	−0.0712401	2E−13						
**Not Supported DI**	−0.282038394	9E−194	0.5624197	0	−0.0179591	0.064						
**Unclear DI**	−0.123288782	3E−37	−0.0874223	2E−19	0.369684	0						

S KO, supported in keyword occurrence; NS KO, not supported in keyword occurrence; U KO, unclear in keyword occurrence; S DI, supported in dominant instances; N DI, not supported in dominant instances; U DI, unclear in dominant instances. Black cells were not logically eligible for correlation.

### Refined dataset

The results displayed in this section have all met the minimum *p*-value threshold of 0.05 and an association strength of at least 0.01, ensuring the statistical significance and reliability of the correlations reported.

**Tables 7**, **8** present the correlations between various topics and the sentiments derived from the keyword occurrence and dominant instance analysis. This cross-referencing allows for the identification of the strength of association between specific topics and the overall support or lack of support for cannabis use as indicated by the studies. Each topic represents the median of a substantial collection of individual keywords. Color coding is referenced in **Tables 1**, **2** in Methodology.

**Table 7:** Topic correlations with the sentiment analyses by keyword occurrence (KO).

Topics KO	Supported	SO *p*	Not supported	NO *p*	Unclear	UO *p*
Anticarcinogenic	0.088297	7.17E−20			−0.02185	0.024209
Anti-inflammatory	0.07737	1.33E−15				
Appetite	0.066646	5.91E−12	0.034275	0.000406		
Cancerous	0.029746	0.002149				
Cancers	0.101808	6.41E−26			−0.0196	0.043147
Chemotherapy	0.087653	1.33E−19	0.052816	5E−08		
Inflammatory	0.061229	2.59E−10				
Nausea	0.079138	2.95E−16	0.046553	1.55E−06		
Opioids	0.056888	4.3E−09			0.062561	1.05E−10
Pain	0.155865	7.53E−59	0.049963	2.52E−07	0.065035	1.88E−11
Radiation therapy	0.026018	0.007273				
Therapeutic	0.481267	0	0.065205	1.67E−11	0.04479	3.8E−06
Tumor growth	0.037471	0.000111				
Tumor size	0.021667	0.025414				
Remission			0.036993	0.000135	0.053688	3E−08

SO p, supported by keyword occurrence p-value; NO p, not supported by keyword occurrence p-value; UO p, Unclear by keyword occurrence p-value. Black cells did not qualify by either minimum r or p-values.

**Table 8:** Topic correlations with the sentiment analyses by dominant instances (DI).

Topics DI	Supported Dom	SD *p*	Not supported Dom	ND *p*	Unclear Dom	UD *p*
Anticarcinogenic	0.057675	2.62E−09				
Anti-inflammatory	0.053567	3.22E−08				
Appetite	0.032948	0.000676				
Cancers	0.080107	1.28E−16	−0.03343	0.000562		
Chemotherapy	0.046437	1.65E−06			−0.02305	0.017419
Immune therapy	0.02196	0.023497				
Inflammatory	0.051397	1.13E−07	−0.02809	0.003762		
Nausea	0.0371	0.000129				
Pain	0.06934	8.04E−13	−0.03394	0.000462		
Therapeutic	0.248491	1.8E−149	−0.11613	2.82E−33	−0.04747	9.62E−07
Tumor growth	0.040946	2.39E−05				

SD p, supported by dominant instances p-value; ND p, Not supported by dominant instances p-value; UD p, Unclear by dominant instances p-value. Black cells did not qualify by either minimum r or p-values.

#### Health metrics results

Focusing on the role of inflammation and therapeutic interventions in cancer progression and treatment outcomes, with an emphasis on how these factors influence patient survival and quality of life. In **Appendix A**, **Table 8** cross references the topic correlations with the sentiment analyses by keyword occurrence, while **Table 9** in **Appendix A**, cross references topic correlations with the sentiment analyses by dominant instances, and **Table 10** in **Appendix A** provides further information on the individual keywords that constitute the different topics.

#### Cancer treatment results

The effectiveness of cannabis in managing cancer-related symptoms like appetite loss, pain, and nausea, as well as its interaction with standard treatments such as chemotherapy and immunotherapy, is examined. In **Appendix A**, **Table 11** cross references the topic correlations with the sentiment analyses by keyword occurrence, while **Table 12** in **Appendix A** cross references topic correlations with the sentiment analyses by dominant instances, and **Table 13** in **Appendix A** provides further information on the individual keywords that constitute the different topics.

#### Cancer dynamics results

The impact of cannabis on cancer progression, including anticarcinogenic effects, tumor growth, size, and remission rates, is investigated to understand its potential as a complementary treatment in oncology. In **Appendix A**, **Table 14** cross references the topic correlations with the sentiment analyses by keyword occurrence, while **Table 15** in **Appendix A** cross references topic correlations with the sentiment analyses by dominant instances, and **Table 16** in **Appendix A** provides further information on the individual keywords that constitute the different topics.

**Figure 3:**
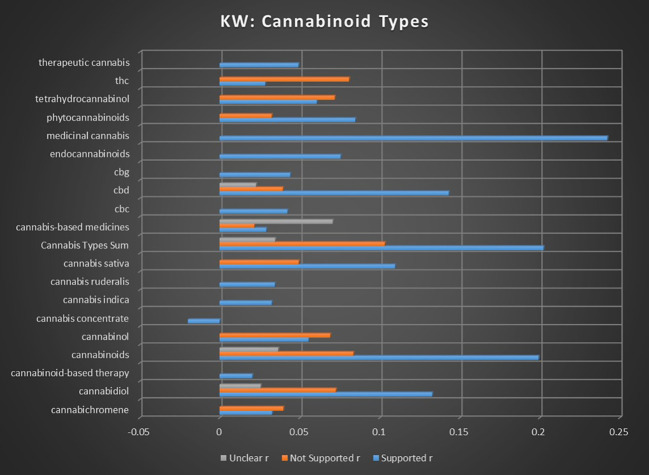
Correlation between individual keywords relating to cannabinoid types and the keyword occurrence association with supported, not supported, and unclear sentiments. All associations meet minimum p-value and r scores for inclusion in refined data.

In addition to the correlations of the preceding topics of health metrics, cancer treatments, and cancer dynamics, a refined dataset was created to explore the keywords associated with specific types of cannabis studied across various research articles, as seen in **Table 17** in **Appendix A** and in **Figure 3** below. This information was deemed relevant to identify any confounders based on endocannabinoids or disrupting outliers, none of which were identified. While differentiating between types of cannabis is not the primary focus of this meta-analysis, detailed information on this subject is provided in **Appendix A**. This **Supplementary Material** is available for researchers interested in delving deeper into the specific impacts and characteristics of different cannabis strains and their relevance to the broader findings of this study.

## Analysis

Cancer, with its profound implications for the lives of patients and viability of healthcare systems, is a condition where the potential palliative and therapeutic benefits of cannabis need to be examined rigorously. The categories analyzed relate to the health metrics involved in measuring the palliative role of cannabis in combination with cancer treatments, as well as cannabis’ directly anticarcinogenic potential.

**Figure 4:**
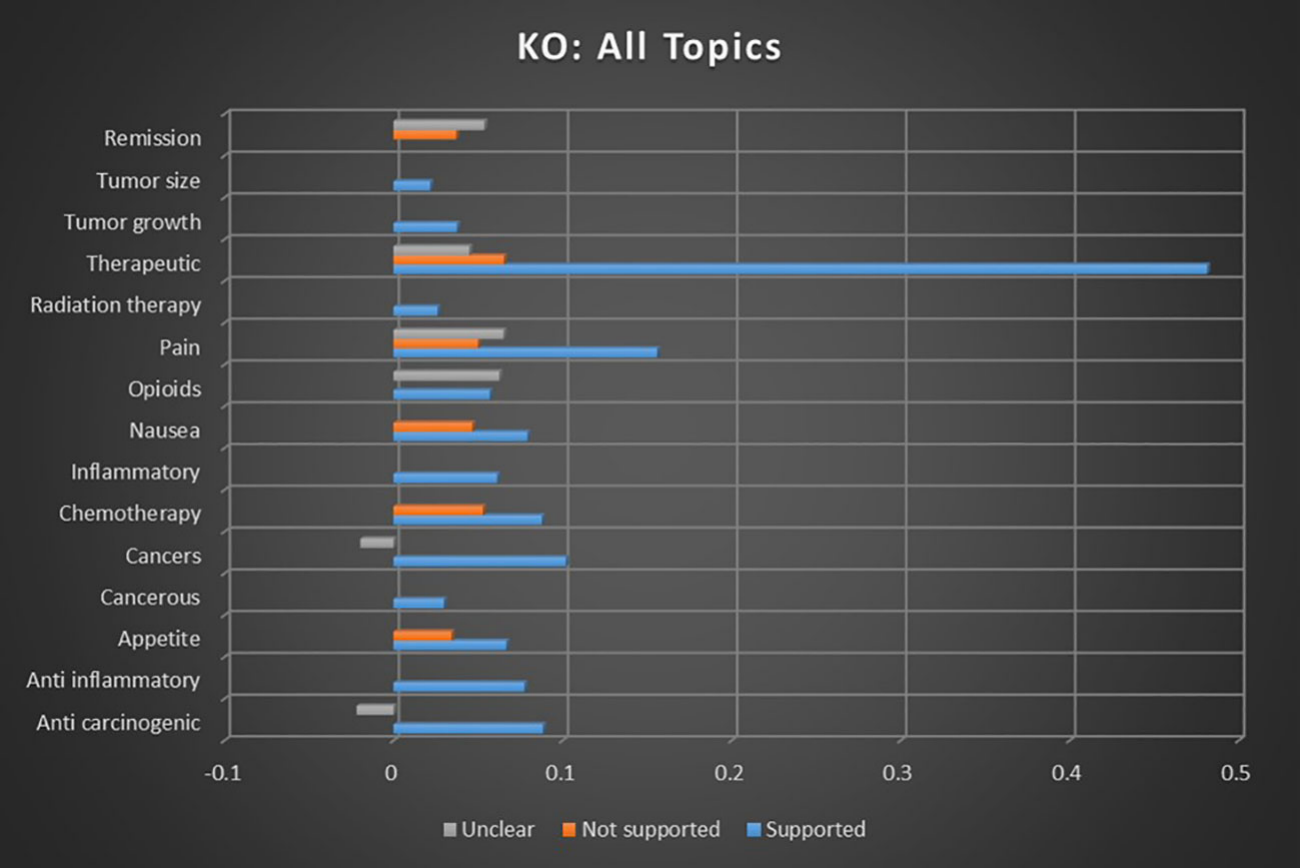
Correlation between all topics and the keyword occurrence association with supported, not supported, and unclear sentiments. All associations meet minimum p-value and r scores for inclusion in refined data.

**Figure 5:**
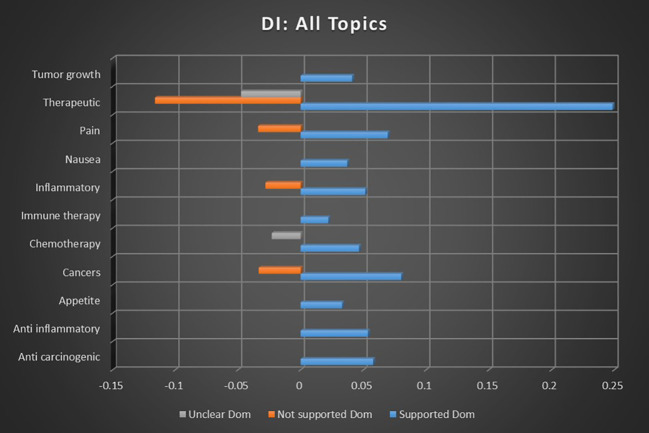
Correlation between all topics and the dominant instance association with supported, not supported, and unclear sentiments. All associations meet minimum p-value and r scores for inclusion in refined data.

When all categories’ sentiment correlations were averaged, combining keyword occurrence in **Figure 4** and dominant instance calculations in **Figure 5**, the correlation strength of cannabis and all cancer topics with supported sentiments was 31.38× stronger than that of not supported sentiments, and 36.79× stronger than with unclear sentiments. The correlation strength of cannabis and all cancer topics with not supported sentiments was 1.17× stronger than that of unclear sentiments.

This analysis indicates that there is a reliable consensus on viability of cannabis as an adjunctive and direct therapeutic intervention for cancer. This meta-analysis supports the potential of cannabis as a beneficial adjunctive treatment in oncology based on the consistency of consensus showing support for the use of medical cannabis both as palliative care during cancer treatment and as a possibly anticarcinogenic/tumorigenic agent.

### Health metrics analysis

When all topic sentiment correlations were averaged, combining keyword occurrence and dominant instance calculations, the correlation strength of cannabis and health metrics with supported sentiments was 46.98× stronger than that of not supported sentiments and 120.97× stronger than that of unclear sentiments. The correlation strength of cannabis and health metrics with not supported sentiments was 2.57× stronger than that of unclear sentiments.

The data regarding cannabis and its relation to health metrics, particularly inflammation and biomarkers, indicate a strong and consistent consensus supporting the anti-inflammatory and therapeutic potential of cannabis. In the analysis of keyword occurrences, no significant negative or unclear sentiments were found related to the inflammatory or anti-inflammatory topics, and dominant instances revealed an inverse correlation between not supported sentiments and the inflammatory topic. This suggests that studies focused on inflammation are significantly more likely to report benefits rather than risks, indicating a consensus on cannabis’ anti-inflammatory effects.

The therapeutic use of cannabis emerges as the most prominent topic within the health metrics category, continuing the pattern observed in inflammation-related studies. The data indicate that studies investigating therapeutic cannabis use are overwhelmingly likely to present supported sentiments, with inverse relationships between not supported and unclear sentiments. This combination indicates that not only are studies relating to cannabis and health metrics more likely than average to result in supporting outcomes, they are less likely than average to result in opposing or unclear outcomes. This strengthens the evidence of a robust consensus in favor of therapeutic cannabis.

Most health metrics keywords reflect a strong supported sentiment, with the exceptions of “chronic inflammatory process” and “EPO.” The slight predominance of not supported sentiments for chronic inflammatory processes may reflect a gap in knowledge and an ongoing debate regarding the specific mechanisms of cannabinoids in the inflammatory system. The inverse relationship between erythropoietin (EPO) and supported outcomes suggests potential concerns with blood platelet interactions in studies involving this biomarker. This may indicate an area of further investigation to answer safety questions in platelet-specific contexts.

#### Topic

**Figure 6:**
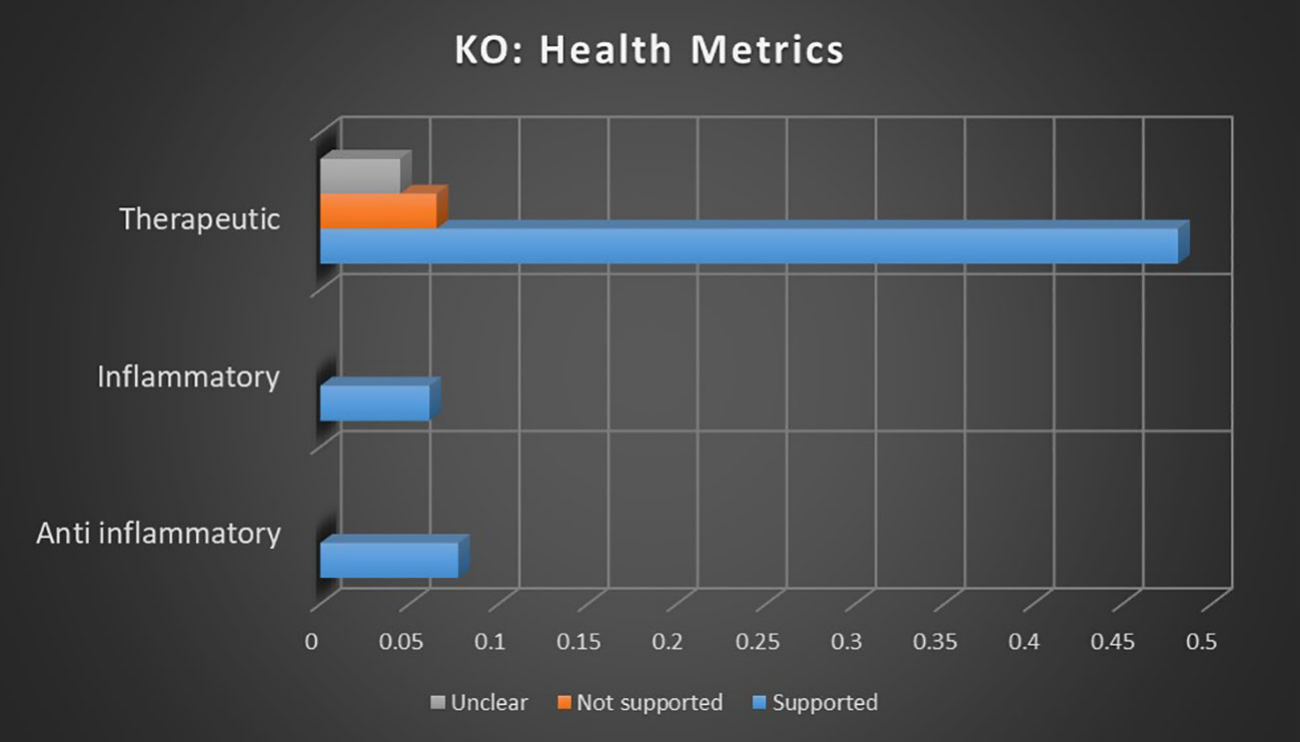
Correlation between topics relating to cannabis and health metrics and the keyword occurrence association with supported, not supported, and unclear sentiments. Horizontal axis measures Pearson’s r strength. All associations meet minimum p-value and r scores for inclusion in refined data.

**Figure 7:**
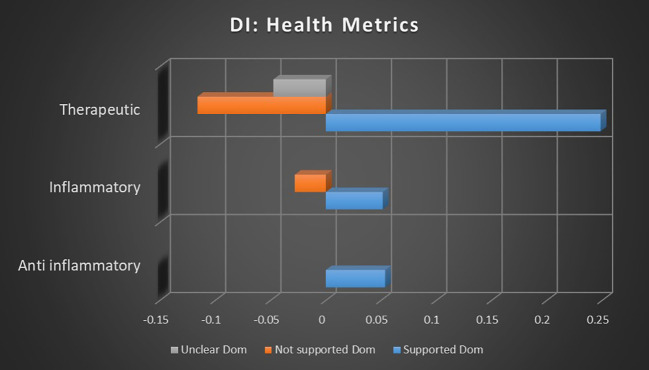
Correlation between topics relating to cannabis and health metrics and the dominant instance association with supported, not supported, and unclear sentiments. Horizontal axis measures Pearson’s r strength. All associations meet minimum p-value and r scores for inclusion in refined data.

Comparing the association strengths of topics with supported, not supported, and unclear sentiments can suggest the strength of potential consensus. As seen in **Figure 6**, for the primary focus of keyword occurrences, there were no negative or unclear sentiments related to the inflammatory or anti-inflammatory topics that met minimum significance thresholds. Analyzing dominant instances in **Figure 7** reveals an inverse correlation between the not supported sentiment and the inflammatory topic, indicating that studies involving inflammation were less likely than average to find the risks outweighed the benefits. This suggests a very likely consensus supporting the anti-inflammatory potential of therapeutic cannabis.

The therapeutic topic is the largest from the health metrics category and continues the pattern seen in the inflammation topics. When measuring keyword occurrences, studies investigating therapeutic use of cannabis were 638.08% more likely to present supported sentiments than not supported sentiments and 974.49% more likely to present supported than unclear sentiments. Not supported sentiments were 45.57% more likely than unclear sentiments. Dominant instance calculations report an inverse relationship between not supported and unclear sentiments and the topic of therapeutic cannabis, indicating that those sentiments are less likely than average for that topic. This suggests that there is a consistent consensus in support of therapeutic cannabis.

#### Keywords

**Figure 8:**
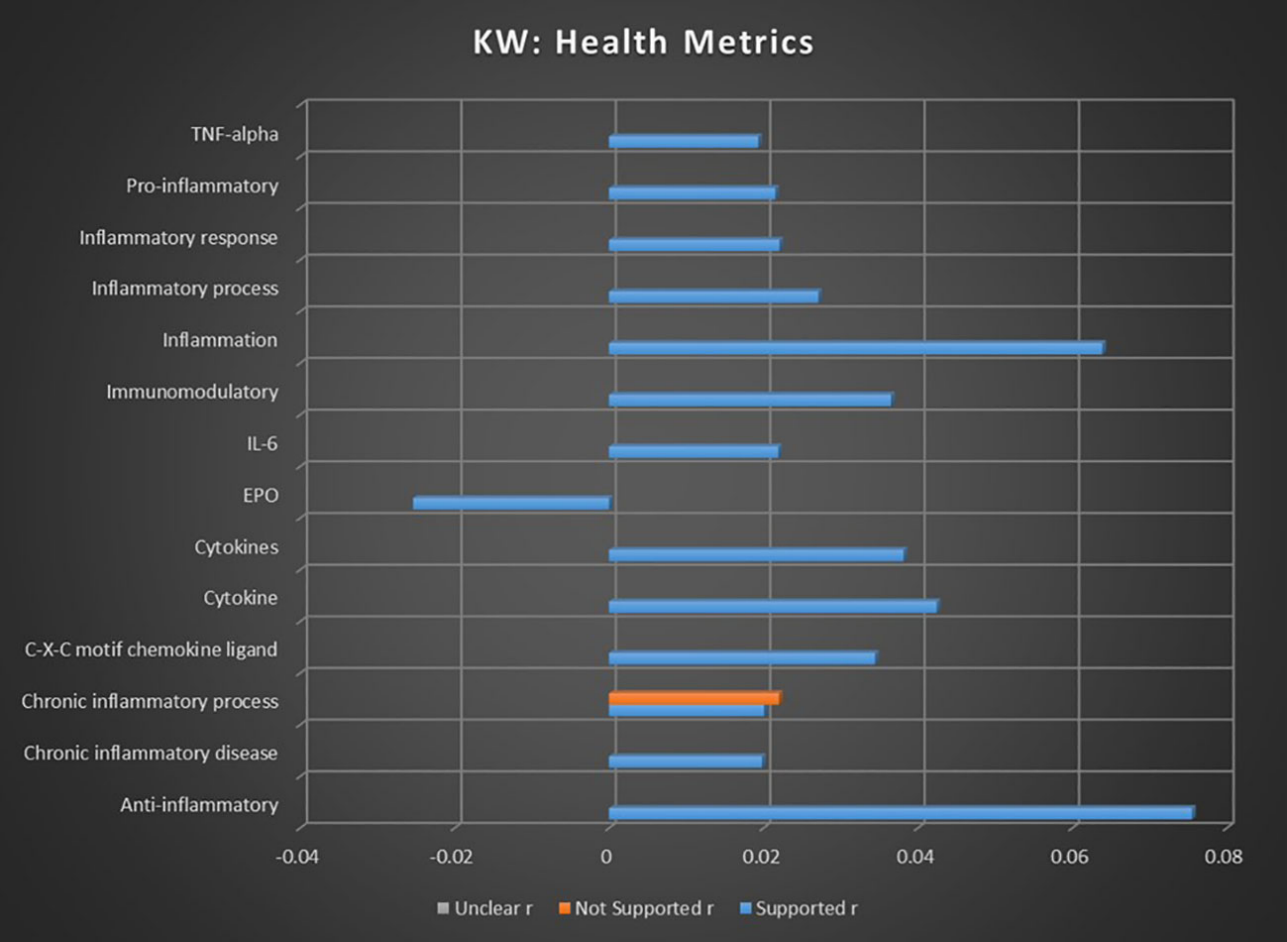
Correlation between individual keywords relating to cannabis and health metrics and the keyword occurrence association with supported, not supported, and unclear sentiments. Horizontal axis measures Pearson’s r strength. All associations meet minimum p-value and r scores for inclusion in refined data.

As seen in **Figure 8**, the majority of all health metrics keywords reflect an overwhelming supported sentiment, with only two exceptions.

The keyword of chronic inflammatory process demonstrated a not supported association strength that was very slightly higher than the supported association strength, potentially reflecting disagreement relating to the mechanisms and processes of cannabinoids with the inflammatory system.

The keyword “EPO” reported an inverse relationship with supported outcomes, suggesting that studies involving EPO metrics were less likely to coincide with studies supporting the use of cannabis. It is possible this outlying biomarkers may be a result of highly complex interactions with both the endocannabinoid system and cancer (29).

A potentially valuable finding is the consistent trend of biomarkers and inflammatory metrics that show strong preponderance for supported sentiments, as this indicates the frequent presence of quantifiable biological factors with consistent reporting patterns. Given the frequent issues involving uncertainty and efficacy surrounding medical cannabis, a scientific consensus on the viability of inflammatory biomarkers is notable.

### Cancer treatment analysis

When all topic sentiment correlations were averaged, combining keyword occurrence and dominant instance calculations, the correlation strength of cannabis and cancer treatments with supported sentiments was 10.93× stronger than that of not supported sentiments and 3.21× stronger than that of unclear sentiments. The correlation strength of cannabis and cancer treatments with unclear sentiments was 3.41× stronger than that of not supported sentiments.

The analysis of the data regarding cannabis as a palliative adjunct to cancer treatments reveals a generally positive trend in the sentiment surrounding its use, with varying degrees of consensus depending on the specific application. Research into the role of cannabis in managing appetite shows strong support, with studies significantly favoring the benefits of cannabis in improving appetite. Similarly, the use of cannabis in conjunction with chemotherapy demonstrates a consistent trend toward supported outcomes, although the degree of consensus varies, suggesting some debate in the field. For immune therapy and radiation therapy, the data are less conclusive, with only modest associations with supported sentiments, indicating gaps in knowledge requiring further research to clarify these potential roles of cannabis.

In terms of symptom management, such as nausea and pain, cannabis appears to have a reasonable level of support, especially in pain management where the consensus is notably stronger. However, the data regarding the use of cannabis as an alternative or complement to opioid analgesics are more complex, with a significant presence of unclear sentiments, reflecting an ongoing controversy and the need for more refined studies in this area. Overall, the data suggest a consensus in favor of using cannabis as a palliative adjunct in cancer treatment, particularly in relation to chemotherapy side effects and symptom management, although the strength of this consensus varies across different topics, highlighting areas where further research is necessary to solidify these findings.

The consistent pattern among cannabis and cancer treatments suggests a reasonable consensus that the benefits of medical cannabis outweigh the risks. The topics that qualified for the refined dataset relate to appetite, chemotherapy, immune therapy, nausea, opioids, and pain. Where there are multiple sentiments with either a positive or inverse association, comparative strength of association will be provided.

#### Topic

Improvement in appetite, relevant to cancer-related anorexia cachexia syndrome and other eating disorders, is one of the more common interventions considered for medical cannabis. The findings seem to support this, as supported sentiments were 94.44% more likely than not supported sentiments in studies related to medical cannabis and appetite. Dominant instance calculation reported a somewhat weaker association strength, but no significant not supported or unclear sentiments. These factors, taken together, suggest a very likely consensus in support of medical cannabis in relation to appetite improvements.

**Figure 9:**
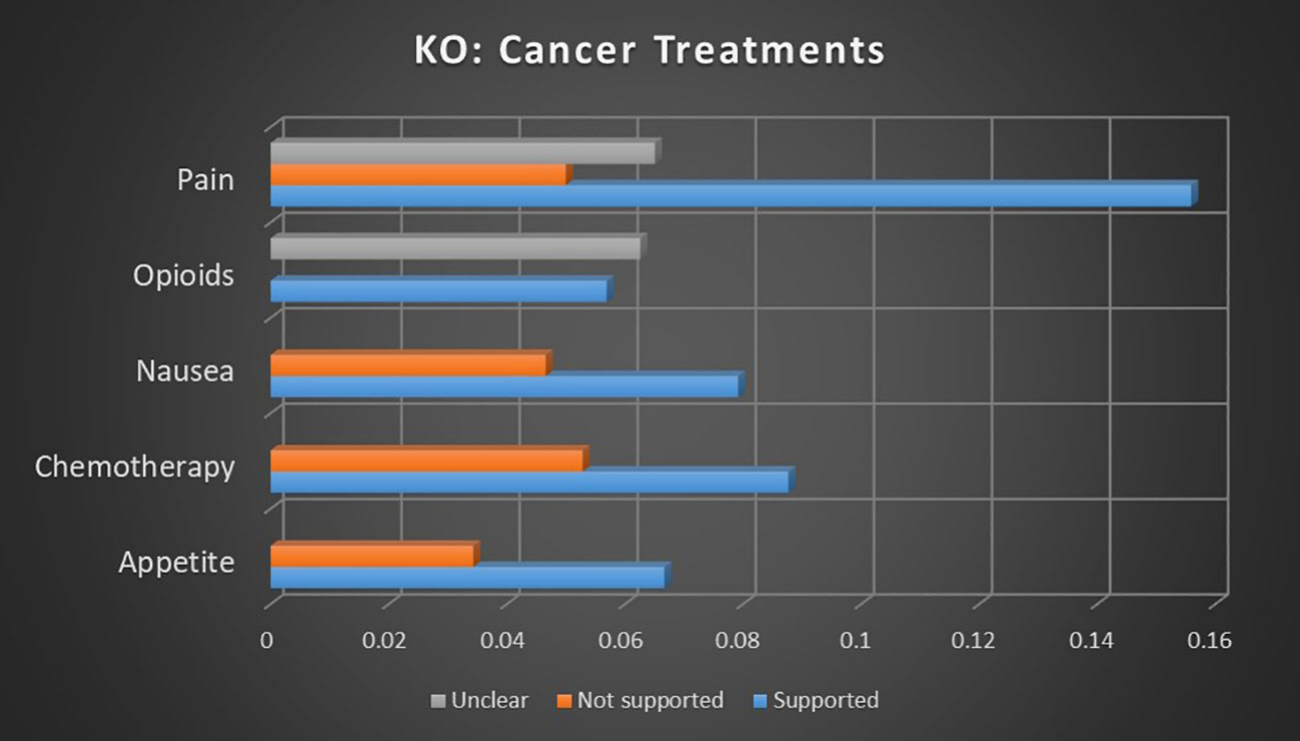
Correlation between topics relating to cannabis and cancer treatments and the keyword occurrence association with supported, not supported, and unclear sentiments. Horizontal axis measures Pearson’s r strength. All associations meet minimum p-value and r scores for inclusion in refined data.

**Figure 10:**
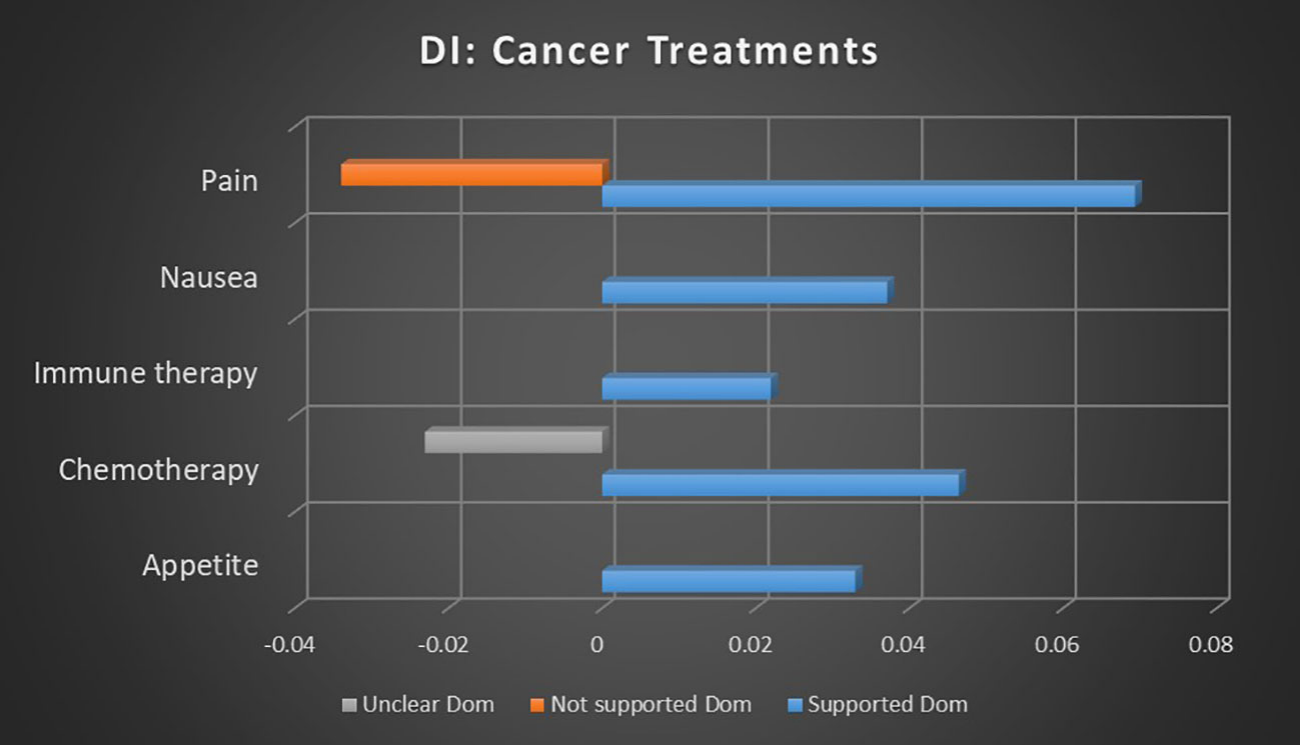
Correlation between topics relating to cannabis and cancer treatments and the dominant instance association with supported, not supported, and unclear sentiments. Horizontal axis measures Pearson’s r strength. All associations meet minimum p-value and r scores for inclusion in refined data.

Chemotherapy is one of the most commonly referenced uses for medical cannabis, but demonstrated mixed results in this meta-analysis. In keyword occurrence calculations as seen in **Figure 9**, there was relatively strong association strengths for both supported and not supported sentiments, with supported sentiments being 65.95% more likely than not supported. Dominant instances, as seen in **Figure 10**, reported more conclusive results, with relatively strong association strength to supported results, no significant not supported results, and unclear sentiment having an inverse relationship, suggesting a much stronger tendency toward supported sentiment with little uncertainty. The combination of these findings suggests a consensus that there is a consensus supporting the use of medical cannabis for chemotherapy, though there is significant debate on the topic.

Dominant instance calculation demonstrated a modest supported sentiment association strength for immune therapy and cannabis, but the absence of a stronger association or other sentiments makes analysis difficult. The same is true for radiation therapy in keyword occurrence calculations.

Nausea is another topic that has historically been closely linked to medical cannabis, though support is not as strong as seen in relation to appetite. Research into medical cannabis and nausea are 69.99% more likely to report supported sentiments than not supported sentiments, according to keyword occurrence calculations. Dominant instances show a weaker association strength for supported sentiment, but no significant not supported or unclear sentiments were present. These findings suggest the reasonable likelihood of a consensus supporting medical cannabis for nausea.

Opioids are a complex but compelling topic related to medical cannabis. The increasing use of cannabis for palliative care, especially pain management, has made it a natural competitor to opioid analgesics (30). Studies involving the topic of opioids and medical cannabis showed that unclear sentiments were virtually equal, with unclear sentiments being 9.97% more likely than supported sentiments. These findings indicate considerable confusion and contradiction at higher levels of analysis.

The topic of pain was a large cohort and represents a growing application for medical cannabis. When calculated through keyword occurrences, medical cannabis in association with pain management is 211.96% more likely to produce supported sentiments than not supported sentiments, and 139.66% more likely than unclear sentiments. Not supported sentiments are 30.16% more likely than unclear sentiments. Dominant instances supported this pattern more clearly, with relatively robust strength in supported sentiment, while not supported sentiments were inversely related to medical cannabis and pain. This indicates that studies involving medical cannabis and pain were significantly more likely than average to result in supported sentiment and significantly less likely than average to report not supported sentiments. This suggests a reliable consensus that the benefits of medical cannabis for pain management outweigh the risks.

#### Keywords

**Figure 11:**
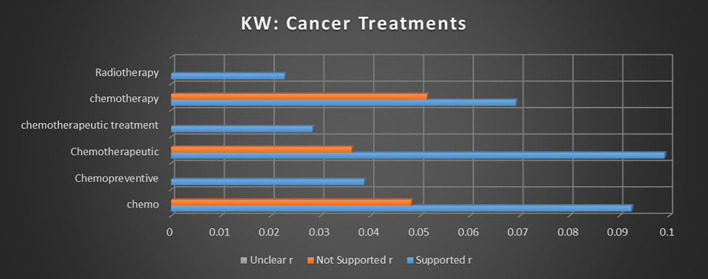
Correlation between individual keywords relating to cannabis and cancer treatments and the keyword occurrence association with supported, not supported, and unclear sentiments. Horizontal axis measures Pearson’s r strength. All associations meet minimum p-value and r scores for inclusion in refined data.

The keyword “chemo” encompasses a broad range of terms related to chemotherapy and related treatments. As seen in **Figure 11**, the analysis indicates a relatively strong correlation between cannabis and “chemo” with supported sentiments, being 191.75% more likely than not supported sentiments. This substantial difference suggests a solid consensus in favor of using medical cannabis as an adjunct to chemotherapy, highlighting its potential therapeutic role in enhancing the effectiveness or mitigating the side effects of chemotherapy.

The keyword “chemopreventative” demonstrated a modest association strength with supported sentiments, with no significant not supported or unclear sentiments reported. Although the association strength is not particularly high, the absence of significant opposing sentiments suggests that there is potential for a developing consensus in support of cannabis’ chemopreventative properties, particularly in its role in preventing cancer progression or recurrence.

The keyword “chemotherapeutic” showed a continuation of the pattern observed with “chemo,” with a strong association with supported outcomes. Cannabis and chemotherapeutics were found to be 273.18% more likely to be associated with supported sentiments than not supported sentiments. This robust correlation reinforces the evidence from the keyword “chemo,” suggesting a reliable and growing consensus in support of using cannabis as an adjunctive treatment in chemotherapeutic protocols.

The term “chemotherapeutic treatment” reported a modest association with supported sentiments, with no other sentiments meeting the significance thresholds. While the isolated association strength is modest and lacks comparative sentiments, this result, when aggregated with other chemotherapeutic-related terms, reinforces the overall trend of supporting cannabis as a beneficial adjunct in chemotherapy.

The keyword “chemotherapy” represents another variation in the semantic range of cancer treatment terms, continuing the trend toward supported sentiment. Cannabis in conjunction with chemotherapy is 134.8% more likely to coincide with a supported sentiment than a not supported sentiment. This result further strengthens the consistent pattern establishing a broad consensus in support of using cannabis alongside chemotherapy in cancer treatment.

The keyword “radiotherapy” reported a modest association with supported sentiments. However, the small size of the association strength, coupled with the lack of significant not supported or unclear sentiments, makes it challenging to draw definitive conclusions about the consensus behind cannabis use in radiotherapy. The data suggest a potential trend toward support, but the evidence remains insufficient to confirm a strong consensus.

### Cancer dynamics analysis

When all topic sentiment correlations were averaged, combining keyword occurrence and dominant instance calculations, the correlation strength of cannabis and cancer with supported sentiments was 32.40× stronger than with not supported sentiments and 14.14× stronger than with unclear sentiments. The correlation strength of cannabis and cancer with unclear sentiments was 2.29× stronger than with not supported sentiments.

The category of cannabis and cancer involves one of the most rapidly expanding fields of study related to medical cannabinoids. Although cannabis has long been used to ameliorate the adverse effects of chemotherapy during cancer treatment, there has been an increasing focus on using cannabis directly as an anticarcinogenic intervention. The examination of cannabis as a direct cancer treatment, particularly in its role as an anticarcinogenic agent, represents a rapidly evolving field within medical research.

This meta-analysis reveals that the majority of topics related to cancer dynamics demonstrate a strong association with sentiments supporting the use of medical cannabis. Notably, the anticarcinogenic potential of cannabis shows robust support, with no significant findings to the contrary, suggesting a reliable consensus in this area. However, certain topics, such as remission, present weaker or unclear associations, indicating either a lack of sufficient research or inconclusive results in this context. Overall, the data suggest a solidifying consensus around the use of cannabis in cancer treatment, particularly in reducing tumor growth and addressing various oncological conditions, though some areas still require further investigation to fully understand the therapeutic potential of cannabis in oncology.

The topics that met refined dataset thresholds include anticarcinogenic, cancerous, cancers, remission, tumor growth, and tumor size. Where there are multiple sentiments with either a positive or inverse association, comparative strength of association will be provided.

#### Topic

**Figure 12:**
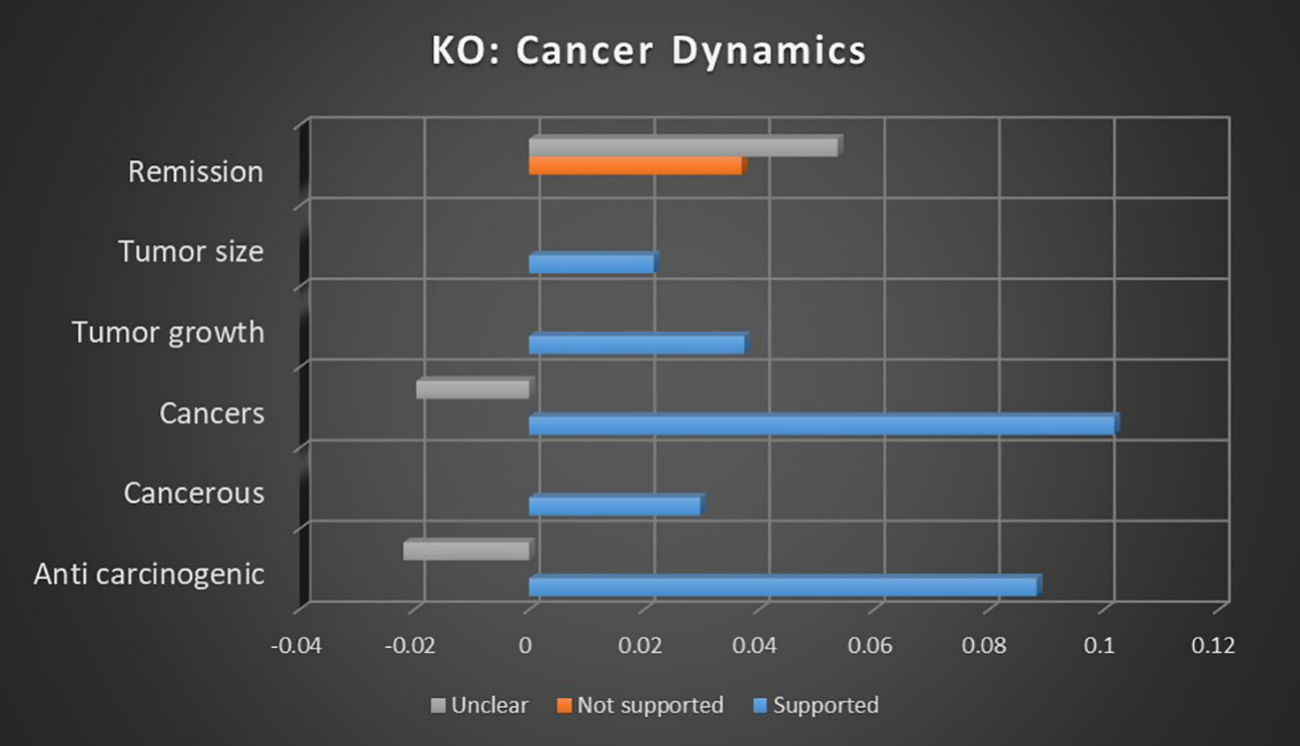
Correlation between topics relating to cannabis and cancer dynamics and the keyword occurrence association with supported, not supported, and unclear sentiments. Horizontal axis measures Pearson’s r strength. All associations meet minimum p-value and r scores for inclusion in refined data.

**Figure 13:**
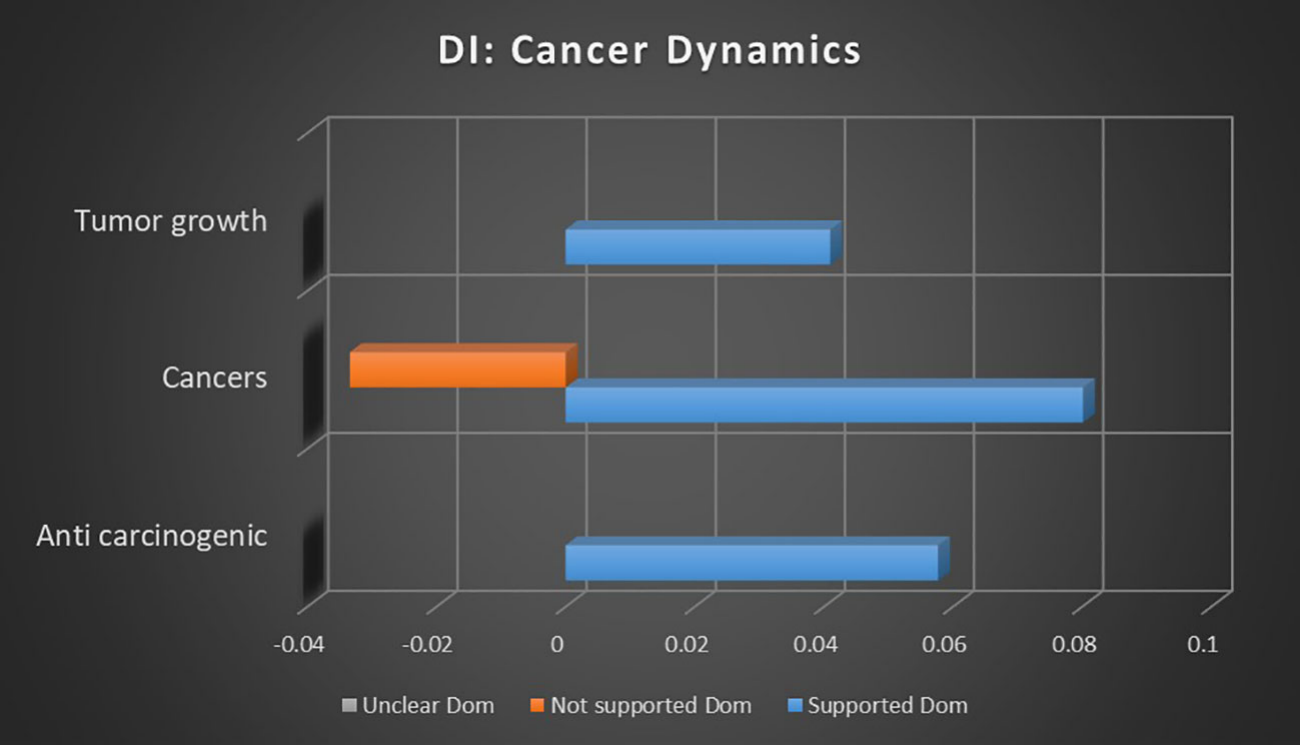
Correlation between topics relating to cannabis and cancer dynamics and the dominant instance association with supported, not supported, and unclear sentiments. Horizontal axis measures Pearson’s r strength. All associations meet minimum p-value and r scores for inclusion in refined data.

Given the high-profile nature of this recent area of research, the association strength of anticarcinogenic research into medical cannabis is surprising. **Figures 12**, **13** reveal that both keyword occurrences and dominant instance calculations showed relatively strong association with supported sentiments, with keyword occurrences demonstrating supported sentiments being 304.13% more likely than unclear sentiments. In neither calculation were any significant not supported sentiments found.

The topic of cancerous growths in relation to medical cannabis reported a modest supported sentiment association, with no significant not supported or unclear sentiments. Without a stronger association strength or contrasting not supported or unclear sentiments, it is difficult to make a meaningful analysis on this topic.

The topic of cancers and medical cannabis is far more easily discerned. In keyword occurrence calculations there is a relatively strong association with supported sentiments, while there's an inverse relationship among unclear sentiments. Dominant instance calculation showed a comparably strong supported sentiment, but with not supported sentiments showing an inverse correlation to the topic of cancers. Taken together, these findings suggest that the study of cancers has a consistent consensus in support of medical cannabis, with not supported or unclear sentiments being even less likely than average.

The topic of remission in keyword occurrence methodology is an outlier. There was no significant supported sentiment reported, and unclear sentiments are 45.13% more likely than not supported sentiments. This suggests a topic that is either relatively untested or arguably debunked. Given the relative newness of cannabis cancer treatments, the former is more likely.

Both keyword occurrence and dominant instance methodologies reported tumor growth to have an association with supported sentiments, with no significant not supported or unclear sentiments found. The association strength is moderate but enough to suggest the potential or nascent consensus on the viability of medical cannabis for treating tumor growth.

Keyword occurrence calculation demonstrated a modest supported sentiment association strength for tumor size and cannabis, but the absence of a stronger association or other sentiments makes analysis difficult.

#### Keywords

**Figure 14:**
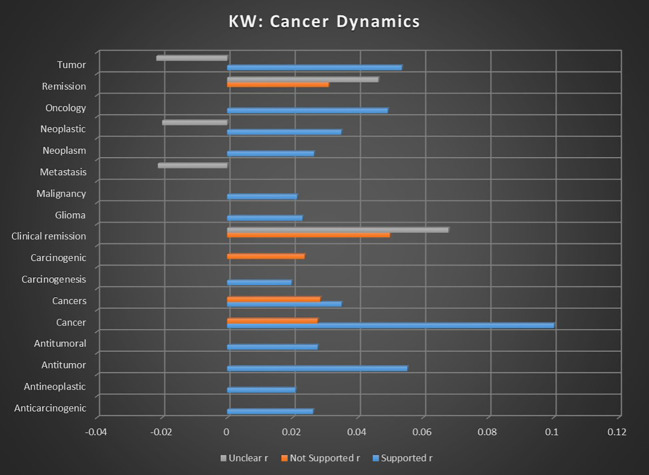
Correlation between individual keywords relating to cannabis and cancer dynamics and the keyword occurrence association with supported, not supported, and unclear sentiments. Horizontal axis measures Pearson’s r strength. All associations meet minimum p-value and r scores for inclusion in refined data.

Keyword occurrence calculation for individual keywords related to cancer dynamics showed some of the most consistent trends in any category studied in this meta-analysis, as illustrated in **Figure 14**. Only for the keyword cancers was the not supported sentiment comparable, with supported sentiments being 22.93% more likely than not supported sentiments.

The only keywords that did not show a tendency toward supported sentiment were remission and clinical remission, echoing the trend in the Topic analysis. In neither keyword were any significant supported sentiments reported, with unclear sentiments being more common than not supported sentiments by 48.84% for remission and by 35.96% for clinical remission. This suggests a lack of consensus, though with a sizable body of evidence in opposition to the idea of cannabis for cancer remission.

The keyword “cancer,” encompassing a wide range of meanings, presented a useful contrast between supported and not supported sentiments. Sentiments in support of medical cannabis had a prevalence of 361.68% in comparison to sentiments in opposition, suggesting a potent consensus. This may be considered a microcosm of the broader topic of cannabis and cancer.

For the terms related to antitumor, oncology, neoplastic, and tumor, relatively strong association strengths were combined with a lack of significant not supported sentiments and/or inverse unclear sentiments. Taken together, these factors suggest areas that are experiencing consistent consensus on the support of medical cannabis for those terms, with less uncertainty than usual.

## Conclusion

### Limitations

Although efforts have been made to ensure as rigorous a methodology as possible, with strict *p*-values, correlation coefficient minimums, and a sensitivity analysis, any sentiment analysis will inevitably include inaccuracies, in this case additionally dependent on machine learning. Broad correlation patterns should not be considered precise or exhaustive, even if they accurately predict trends. Correlational trends are imperfect numerical representations of complex qualitative information and cannot be used to precisely predict effects or efficacy on an individual scale.

### Discussion

Given the heterogeneity and inconsistent history of medical cannabis research, it was determined that a large-scale meta-analysis was necessary to identify where there was scientific consensus and where there were gaps in the understanding of the current state of research on medical cannabis, particularly in its applications within oncology. This work constitutes a data-driven sentiment analysis and comprehensive correlational calculations through two separate methodologies, reviewing the text from 10,641 peer-reviewed studies on medical cannabis to determine the consensus on whether the benefits outweigh the risks for various aspects of cancer care. The aggregated correlation strength of cannabis across all cancer topics indicates that support for medical cannabis is 31.38× stronger than opposition to it.

These findings revealed a significant trend suggesting support of cannabis’ therapeutic potential, particularly in managing cancer-related symptoms and possibly exerting direct anticarcinogenic effects. Across all categories examined—health metrics, cancer treatments, and cancer dynamics—there is a consistent consensus that supports the potential of medical cannabis.

The anti-inflammatory properties of cannabis were strongly supported, with a robust consensus indicating that cannabis’ benefits in reducing inflammation significantly outweigh potential risks. This is a critical finding, as inflammation is a key factor in many chronic diseases, including cancer. Extensive biomarkers support the idea that cannabis has a broad utility in managing various health conditions, with minimal significant opposition or ambiguity in the data. Cancer treatments, including chemotherapy, appetite management, and pain relief, also indicated strong support for the use of cannabis as an adjunct therapy, though there is some variability in the strength of this consensus across different applications. The analysis also revealed particularly strong support for cannabis’ potential as an anticarcinogenic agent. The robust association between cannabis and reduced tumor growth, as well as its potential to influence cancerous processes, highlights an emerging consensus within the scientific community.

### Implications

The findings of this meta-analysis have significant implications for both public health research and medical practice. First and foremost, the strong consensus supporting the therapeutic use of cannabis, particularly in the context of cancer, suggests that there is a substantial scientific basis for re-evaluating cannabis’ legal status and its classification as a Schedule I substance. The data presented here indicate that cannabis has a well-established role in managing symptoms related to cancer and may have both direct and indirect anticancer properties, which challenges the notion that it has no accepted medical use.

The meta-analysis also identified knowledge gaps where further research could have disproportionate benefits. The precise mechanisms of cannabinoid interaction with inflammatory processes appears to lack clear consensus, as does the topic of cannabis and opioid use in pain management. Furthermore, despite consensus around cannabis’ anticarcinogenic potential, there is significant aversion to any linkage with remission, suggesting an area requiring further clarification. Inverse supported correlations with the marker EPO indicate a topic that may pose a safety concern.

By identifying gaps in the current research and areas of strong consensus, this meta-analysis provides a roadmap for future studies that could clarify cannabis’ place in the medical toolkit. Additionally, the evidence supporting cannabis’ anti-inflammatory properties suggests broader applications beyond oncology, potentially influencing the treatment of other chronic inflammatory diseases.

In medical practice, the strong support for cannabis as a palliative adjunct to cancer treatments offers healthcare providers a data-driven foundation to consider cannabis as part of a comprehensive cancer care strategy. The demonstrated efficacy in managing symptoms like pain, nausea, and appetite loss can significantly enhance patients’ quality of life, making cannabis a valuable tool in both palliative care and potentially in curative settings. The growing consensus around cannabis’ therapeutic benefits also highlights the need for medical professionals to stay informed about the latest research, as cannabis continues to evolve from a controversial substance to a scientifically validated treatment option.

This meta-analysis quantifies the consensus on the therapeutic potential of medical cannabis, and the results challenge the current regulatory assumptions that restrict its use. By providing a detailed examination of the correlations between cannabis use and large-scale research sentiments, particularly in oncology, this study lays the groundwork for future research and policy decisions that could significantly impact public health and patient care.

## Data Availability

The datasets presented in this study can be found in online repositories. The names of the repository/repositories and accession number(s) can be found in the article/**Supplementary Material**.

## References

[1] Cannabis in Medical Practice: A Legal, Historical and Pharmacological Overview of the Therapeutic Use of Marijuana. Mathre ML (Ed.), United Kingdom: McFarland, Incorporated, Publishers (1997).

[2] O’Shaughnessy WB . On the Preparations of the Indian Hemp, or Gunjah: Cannabis Indica Their Effects on the Animal System in Health, and their Utility in the Treatment of Tetanus and other Convulsive Diseases. Prov Med J Retrosp Med Sci. (1843) 5:363–9. PMC5592602 30161735

[3] Gaoni Y Mechoulam R . Isolation, structure, and partial synthesis of an active constituent of hashish. J Am Chem Soc. (1964) 86(8):1646–7. doi: 10.1021/j01062046

[4] Cooper ZD Abrams DI Gust S Salicrup A Throckmorton DC . Challenges for clinical cannabis and cannabinoid research in the United States. J Natl Cancer Inst Monogr. (2021) 2021:114–22. doi: 10.1093/jncimonographs/lgab009 PMC8783595 34850896

[5] Campbell AW . The Medical Marijuana Catch-22: how the federal monopoly on marijuana research unfairly handicaps the rescheduling movement. Am J Law Med. (2015) 41:190–209. doi: 10.1177/0098858815591513 26237987

[6] Marcu J . The legalization of cannabinoid products and standardizing cannabis-drug development in the United States: a brief report. Dialogues Clin Neurosci. (2020) 22:289–93. doi: 10.31887/DCNS.2020.22.3/jmarcu PMC7605018 33162772

[7] Cahill SP Lunn SE Diaz P Page JE . Evaluation of patient reported safety and efficacy of cannabis from a survey of medical cannabis patients in Canada. Front Public Health. (2021) 9:626853. doi: 10.3389/fpubh.2021.626853 34095048 PMC8172603

[8] Troyer J Tanco K . Review of the use of medicinal cannabis products in palliative care. Cancers. (2024) 16:1412. doi: 10.3390/cancers16071412 38611090 PMC11011126

[9] Russo EB . Cannabinoids in the management of difficult to treat pain. Ther Clin Risk Manage. (2008) 4:245–59. doi: 10.2147/tcrm.s1928 PMC2503660 18728714

[10] Lynch ME Campbell F . Cannabinoids for treatment of chronic non-cancer pain; a systematic review of randomized trials. Br J Clin Pharmacol. (2011) 72:735–44. doi: 10.1111/j.1365-2125.2011.03970.x PMC3243008 21426373

[11] MaChado Rocha FC Stéfano SC De Cassia Haiek R Rosa Oliveira LMQ Da Silveira DX . Therapeutic use of Cannabis sativa on chemotherapy-induced nausea and vomiting among cancer patients: systematic review and meta-analysis. Eur J Cancer Care. (2008) 17:431–43. doi: 10.1111/j.1365-2354.2008.00917.x 18625004

[12] Razmovski-Naumovski V Luckett T Amgarth-Duff I Agar MR . Efficacy of medicinal cannabis for appetite-related symptoms in people with cancer: A systematic review. Palliative Med. (2022) 36:912–27. doi: 10.1177/02692163221083437 35360989

[13] Salz T Meza AM Chino F Mao JJ Raghunathan NJ Jinna S . Cannabis use among recently treated cancer patients: perceptions and experiences. Support Care Cancer. (2023) 31:545. doi: 10.1007/s00520-023-07994-y 37650961 PMC10585595

[14] Velasco G Sánchez C Guzmán M . Anticancer mechanisms of cannabinoids. Curr Oncol Rep. (2016) 18:14. doi: 10.3747/co.23.3080 27022311 PMC4791144

[15] Smith LA Azariah F Lavender VT Stoner NS Bettiol S . Cannabinoids for nausea and vomiting in adults with cancer receiving chemotherapy. Cochrane Database Systematic Rev. (2015) 2015:CD009464. doi: 10.1002/14651858.CD009464.pub2 PMC6931414 26561338

[16] De Feo G Case AA Crawford GB Hui D To J Sbrana A . Multinational Association of Supportive Care in Cancer (MASCC) guidelines: cannabis for psychological symptoms including insomnia, anxiety, and depression. Support Care Cancer. (2023) 31:176. doi: 10.1007/s00520-023-07628-3 36809575

[17] To J Davis M Sbrana A Alderman B Hui D Mukhopadhyay S . MASCC guideline: cannabis for cancer-related pain and risk of harms and adverse events [published correction appears in Support Care Cancer. 2023 May 6;31(6):323. doi: 10.1007/s00520-023-07789-1. Support Care Cancer. (2023) 31:202. doi: 10.1007/s00520-023-07662-1 36872397

[18] Zeraatkar D Cooper MA Agarwal A Vernooij RW Leung G Loniewski K . Long-term and serious harms of medical cannabis and cannabinoids for chronic pain: a systematic review of non-randomised studies. BMJ Open. (2022) 12:e054282. doi: 10.1136/bmjopen-2021-054282 PMC9358949 35926992

[19] Lee C Danielson EC Beestrum M Eurich DT Knapp A Jordan N . Medical cannabis and its efficacy/effectiveness for the treatment of low-back pain: a systematic review. Curr Pain Headache Rep. (2023) 27:821–35. doi: 10.1007/s11916-023-01189-0 PMC11095816 38041708

[20] Hanganu B Lazar DE Manoilescu IS Mocanu V Butcovan D Buhas CL . Controversial link between cannabis and anticancer treatments-where are we and where are we going? A systematic review of the literature. Cancers (Basel). (2022) 14:4057. doi: 10.3390/cancers14164057 36011049 PMC9406903

[21] Valenti C Billi M Pancrazi GL Calabria E Armogida NG Tortora G . Biological effects of cannabidiol on human cancer cells: Systematic review of the literature. Pharmacol Res. (2022) 181:106267. doi: 10.1016/j.phrs.2022.106267 35643249

[22] Bachari A Piva TJ Salami SA Jamshidi N Mantri N . Roles of cannabinoids in melanoma: evidence from *in vivo* studies. Int J Mol Sci. (2020) 21:6040. doi: 10.3390/ijms21176040 32839414 PMC7503316

[23] André R Gomes AP Pereira-Leite C Marques-da-Costa A Monteiro Rodrigues L Sassano M . The entourage effect in cannabis medicinal products: A comprehensive review. Pharm (Basel). (2024) 17:1543. doi: 10.3390/ph17111543 PMC11870048 39598452

[24] Mead A . The legal status of cannabis (marijuana) and cannabidiol (CBD) under US law. Epilepsy Behav. (2017) 70:288–91. doi: 10.1016/j.yebeh.2016.11.021 28169144

[25] Almeida CF Teixeira N Correia-da-Silva G Amaral C . Cannabinoids in breast cancer: differential susceptibility according to subtype. Molecules. (2022) 27:156. doi: 10.3390/molecules27010156 PMC8746990 35011388

[26] Sarsembayeva A Schicho R . Cannabinoids and the endocannabinoid system in immunotherapy: helpful or harmful? Front Oncol. (2023) 13:1296906. doi: 10.3389/fonc.2023.1296906 38074691 PMC10699860

[27] Feldman R . Techniques and applications for sentiment analysis. Commun ACM. (2013) 56:82–9. doi: 10.1145/2436256.2436274

[28] Liu B . Sentiment Analysis and Opinion Mining. Switzerland: Morgan & Claypool Publishers (2012).

[29] Gasperi V Evangelista D Savini I Del Principe D Avigliano L Maccarrone M . Downstream effects of endocannabinoid on blood cells: implications for health and disease. Cell Mol Life Sci. (2015) 72:3235–52. doi: 10.1007/s00018-015-1924-0 PMC11113859 25957591

[30] Khalid N Patel P Singh A . Cannabis versus opioids for pain. In: StatPearls. StatPearls Publishing, Treasure Island (FL (2024). Available at: https://www.ncbi.nlm.nih.gov/books/NBK573080/. 34424653

