# Supplementary material for: Meta-analysis of medical cannabis outcomes and associations with cancer
Source: Front Oncol. 2025 Apr 15;15:1490621. doi: 10.3389/fonc.2025.1490621 (PMC12037605; doi:10.3389/fonc.2025.1490621)
Supplement: Supplementary file 1 [file SupplementaryFile1.pdf]

Appendix A

to

Meta-analysis of Medical Cannabis Outcomes and Associations with Cancer

Castle, R., Marzolf, J., Morris, M., Bushell, WC.

Literature review

The following systematic review investigating the potential of cannabis as a treatment for insomnia, anxiety, and depression in cancer patients concluded that there is currently no substantial evidence to support its use for cancer patient symptoms. Citing inconsistent results, the review determined there was no high-quality evidence demonstrating cannabis' efficacy in managing these conditions within the cancer population.

The following systematic review investigating the potential of cannabis as a treatment for insomnia, anxiety, and depression in cancer patients concluded that there is currently no substantial evidence to support its use for cancer patient symptoms. Citing inconsistent results, the review determined there was no high-quality evidence demonstrating cannabis' efficacy in managing these conditions within the cancer population.

Literature review summary

Multinational Association of Supportive Care in Cancer (MASCC) guidelines: cannabis for psychological symptoms including insomnia, anxiety, and depression[i]

A subsequent systematic review built upon the preceding findings, further asserting that cannabinoids do not demonstrate efficacy as an analgesic or viable pain management option for cancer patients. Additionally, the review raised serious concerns about potential adverse events associated with cannabinoid use. These conclusions are somewhat contradicted by the data within the same review, where numerous trials reported positive outcomes in pain management, and no significant incidence rate of serious adverse events was observed.

MASCC guideline: cannabis for cancer-related pain and risk of harms and adverse events[ii]

Further investigation into the adverse effects of medical cannabis involved a systematic review and meta-analysis focusing on its use in chronic pain management. The findings revealed weak evidence supporting the prevalence of significant adverse effects, with serious adverse events being particularly rare. Moreover, the analysis indicated that medical cannabis presents a comparable, if not more favorable, safety profile than other pain management options, such as opioids. This directly contradicts the conclusions of the preceding study that suggested a higher risk of adverse effects, but findings were limited by insufficient data and lack of comparative studies.

Long-term and serious harms of medical cannabis and cannabinoids for chronic pain: a systematic review of non-randomised studies[iii]

While the previous study challenged the notion that cannabis is a dangerous intervention, questions regarding its efficacy as a pain management approach remain. Another study focused on the efficacy of medical cannabis for chronic pain, concluding that cannabis consistently provided pain relief equivalent to opioid analgesics, but without the substance dependence or adverse effects commonly associated with opioids. This systematic review further reported that cannabis use was associated with a decrease in dependence on addictive opioids, suggesting a potentially valuable role for cannabis in reducing opioid reliance in chronic pain management. Again, heterogeneous studies and a lack of narrative consistency limited conclusions.

Medical Cannabis and Its Efficacy/Effectiveness for the Treatment of Low-Back Pain: a Systematic Review[iv]

The contradictory findings regarding adverse effects and analgesic potential of cannabis in systematic reviews prompt further investigation into its broader medical applications. A systematic review was conducted to explore the use of cannabis across various oncological treatments globally. This review identified cannabis as highly regarded in clinical applications for glioblastoma, as an adjunctive treatment with immunotherapy, and in other therapeutic settings. However, despite its popularity and perceived clinical potential, the data collected was found to be inconsistent, preventing the formulation of definitive conclusions.

Controversial Link between Cannabis and Anticancer Treatments-Where Are We and Where Are We Going? A Systematic Review of the Literature[v]

Although the preceding review reported a lack of any consistent findings, clinical popularity is not necessarily indicative of medical efficacy, which was the subsequent systematic review's focus in investigating the biological effects of cannabinoids on human cancer cells. The findings demonstrated that cannabinoids effectively inhibited cancer cell growth, proliferation, and migration, while also exerting anti-inflammatory effects and reducing metastasis. These results suggested that cannabinoids possess potential anti-carcinogenic properties at the cellular level, providing a scientific basis for their therapeutic use in cancer treatment beyond merely palliative care.

Biological effects of cannabidiol on human cancer cells: Systematic review of the literature[vi]

Building on the cellular mechanisms observed in the preceding study, a systematic review focused on the use of cannabinoids in the treatment of melanoma explored various in vivo studies. The review found that cannabinoids demonstrated a capacity to inhibit tumor growth and induce apoptosis in cancer cells, highlighting their potential as a therapeutic option in melanoma. Despite these promising findings, the review noted significant limitations, including the heterogeneity of the studies examined and the absence of a clear consensus on the standard of care involving medical cannabis.

Roles of Cannabinoids in Melanoma: Evidence from In Vivo Studies[vii]

Literature review in detail

Multinational Association of Supportive Care in Cancer (MASCC) guidelines: cannabis for psychological symptoms including insomnia, anxiety, and depression[viii]

1. ***Purpose:*** *The broad goal of this research paper was to systematically review the evidence for the use of cannabis in managing psychological symptoms—specifically anxiety, depression, and sleep disturbances—in cancer patients, with the aim of developing a guideline for its use.*
2. ***Methods:*** *The study involved a literature search of randomized trials and systematic reviews from databases such as MEDLINE, CCTR, EMBASE, and PsychINFO, up until November 12, 2021. The inclusion criteria were randomized controlled trials and systematic reviews that compared cannabis with a placebo or an active comparator specifically for managing psychological symptoms in cancer patients. Two authors independently assessed the studies, and all authors evaluated them for approval.*
3. ***Intervention:*** *The intervention examined was the use of cannabis for managing psychological symptoms (anxiety, depression, and insomnia) in cancer patients. However, the studies varied significantly in their interventions, controls, duration, and outcome measures. Among the 15 randomized controlled trials that met the eligibility criteria, six suggested some benefits of cannabis—five for sleep improvement and one for mood enhancement.*
4. ***Conclusions:*** *The study concluded that there is no high-quality evidence to support the recommendation of cannabis as an intervention for psychological symptoms in cancer patients. The authors emphasized the need for more high-quality research before any definitive guidelines can be established.*
5. ***Limitations:*** *Specific limitations noted include the lack of studies that assessed the efficacy of cannabis on psychological symptoms as primary outcomes in cancer patients. Additionally, the wide variation in interventions, control groups, duration, and outcome measures across the studies makes it difficult to draw definitive conclusions. These limitations highlight the need for more standardized and rigorous research to evaluate the potential benefits of cannabis for psychological symptoms in cancer patients.*

A subsequent systematic review built upon the preceding findings, further asserting that cannabinoids do not demonstrate efficacy as an analgesic or viable pain management option for cancer patients. Additionally, the review raised serious concerns about potential adverse events associated with cannabinoid use. These conclusions are somewhat contradicted by the data within the same review, where numerous trials reported positive outcomes in pain management, and no significant incidence rate of serious adverse events was observed.

MASCC guideline: cannabis for cancer-related pain and risk of harms and adverse events[ix]

1. ***Purpose:*** *The broad goal of the research paper was to perform a systematic review of randomized cannabis trials in cancer patients, specifically to establish a guideline for its use in managing cancer-related pain and to summarize the risks of harm and adverse events associated with its use.*
2. ***Methods:*** *The study involved a systematic review of randomized trials, with or without meta-analysis, that were identified through databases such as MEDLINE, CCTR, Embase, and PsychINFO. The population studied included adult cancer patients, and the trials were randomized, comparing cannabinoids either with a placebo or an active comparator. The quality of the studies was assessed using the Jadad grading system.*
3. ***Intervention:*** *The intervention analyzed was the use of cannabinoids as a treatment for cancer pain. The overall results indicated that, while some trials reported positive primary endpoints, these results could not be consistently reproduced in similar trials. High-quality systematic reviews with meta-analyses found little evidence to support the effectiveness of cannabinoids as an adjuvant or analgesic for cancer pain.*
4. ***Conclusions:*** *The study concluded that the MASCC (Multinational Association of Supportive Care in Cancer) panel recommends against the use of cannabinoids as an adjuvant analgesic for cancer pain. Additionally, the study suggests that the potential risks of harm and adverse events should be carefully considered, especially in cancer patients undergoing treatment with a checkpoint inhibitor.*
5. ***Limitations:*** *Specific limitations include the inconsistency in evidence regarding the types and levels of harm that patients might experience when using cannabinoids. Additionally, the inability to reproduce positive results in similarly designed trials highlights a lack of consistent evidence for the effectiveness of cannabinoids in managing cancer pain. These factors suggest that the findings are not definitive and affirm the need for further research.*

Further investigation into the adverse effects of medical cannabis involved a systematic review and meta-analysis focusing on its use in chronic pain management. The findings revealed weak evidence supporting the prevalence of significant adverse effects, with serious adverse events being particularly rare. Moreover, the analysis indicated that medical cannabis presents a comparable, if not more favorable, safety profile than other pain management options, such as opioids. This directly contradicts the conclusions of the preceding study that suggested a higher risk of adverse effects, but findings were limited by insufficient data and lack of comparative studies.

Long-term and serious harms of medical cannabis and cannabinoids for chronic pain: a systematic review of non-randomised studies [x]

1. ***Purpose:*** *The broad goal of this research paper was to establish the prevalence of long-term and serious harms associated with the use of medical cannabis for chronic pain management.*

2. ***Methods:*** *The study conducted a systematic review and meta-analysis, searching databases such as MEDLINE, EMBASE, PsycINFO, and CENTRAL from their inception to April 1, 2020. The study focused on non-randomized studies reporting the harms of medical cannabis or cannabinoids in adults or children living with chronic pain, with a minimum follow-up period of four weeks. Two independent reviewers screened the search results, extracted data, and assessed the risk of bias. The random-effects models were used for meta-analysis, and the Grades of Recommendations, Assessment, Development, and Evaluation (GRADE) approach was employed to evaluate the certainty of the evidence.*
3. ***Intervention:*** *The intervention analyzed was the use of medical cannabis or cannabinoids for chronic pain management. The overall results suggested that while adverse events are relatively common, with a prevalence of 26.0%, serious adverse events were rare. Adverse events related to mood were the most commonly reported, with a prevalence of 13.5%.*
4. ***Conclusions:*** *The study concluded that there is very low certainty evidence indicating that adverse events are common among people using medical cannabis for chronic pain, particularly psychiatric adverse events. However, the evidence suggests that serious adverse events, including those leading to discontinuation, cognitive adverse events, accidents, injuries, dependence, and withdrawal syndrome, are rare. The study did not find any evidence suggesting medical cannabis compares negatively with other pain management options, such as opioids.*
5. ***Limitations:*** *Specific limitations noted include the very low certainty of the evidence, which reduces the confidence in the findings. Additionally, the studies reviewed were non-randomized, which may introduce bias. The study also highlighted insufficient evidence on how the harms of medical cannabis compare to those of other pain management options, such as opioids, limiting the ability to draw comprehensive conclusions about its safety profile relative to other treatments.*

While the previous study challenged the notion that cannabis is a dangerous intervention, questions regarding its efficacy as a pain management approach remain. Another study focused on the efficacy of medical cannabis for chronic pain, concluding that cannabis consistently provided pain relief equivalent to opioid analgesics, but without the substance dependence or adverse effects commonly associated with opioids. This systematic review further reported that cannabis use was associated with a decrease in dependence on addictive opioids, suggesting a potentially valuable role for cannabis in reducing opioid reliance in chronic pain management. Again, heterogeneous studies and a lack of narrative consistency limited conclusions.

Medical Cannabis and Its Efficacy/Effectiveness for the Treatment of Low-Back Pain: a Systematic Review[xi]

1. ***Purpose:*** *The broad goal of this systematic review was to evaluate the current evidence regarding the efficacy and effectiveness of medical cannabis for treating low back pain (LBP), specifically focusing on pain levels and overall opioid use in individuals prescribed medical cannabis for LBP.*

2. ***Methods:*** *The study conducted searches in databases such as MEDLINE (PubMed), Embase, and CINAHL, covering research published in the past 10 years (2011-2021). The inclusion criteria involved English-language articles that included adult participants with LBP who were prescribed medical cannabis, and who might also be using opioids for their LBP. The Joanna Briggs Institute framework was used for critical appraisal, and both study quality and risk of bias were evaluated. A narrative synthesis approach was employed to analyze the findings.*

3. ***Intervention:*** *The intervention studied was the use of medical cannabis for managing low back pain (LBP) and its potential impact on pain levels and opioid use. Among the 12 studies included in the synthesis, all but one (a randomized controlled trial) indicated a decrease in LBP levels or opioid use over time following medical cannabis use. The randomized controlled trial (RCT) reported no statistically significant difference in LBP between cannabis and placebo groups.*

4. ***Conclusions:*** *The study described a growing interest in medical cannabis as an alternative treatment for low back pain, with mixed quality evidence to support its use as a first-line treatment. Pain management efficacy was approximately equal to opioids and resulted in decreased opioid reliance. All but one study reported a decrease in pain levels or opioid use with cannabis, but unclear findings in the RCT highlights the need for more robust evidence.*

5. ***Limitations:*** *Specific limitations include the minimal high-quality evidence available, as most of the studies included were observational or case studies rather than randomized controlled trials. The review also noted the infancy of research in this area, with a significant need for more rigorous and high-quality studies to establish the efficacy of medical cannabis for LBP. Additionally, the narrative synthesis approach may limit the ability to draw definitive conclusions from the diverse study designs and outcomes.*

The contradictory findings regarding adverse effects and analgesic potential of cannabis in systematic reviews prompt further investigation into its broader medical applications. A systematic review was conducted to explore the use of cannabis across various oncological treatments globally. This review identified cannabis as highly regarded in clinical applications for glioblastoma, as an adjunctive treatment with immunotherapy, and in other therapeutic settings. However, despite its popularity and perceived clinical potential, the data collected was found to be inconsistent, preventing the formulation of definitive conclusions.

Controversial Link between Cannabis and Anticancer Treatments-Where Are We and Where Are We Going? A Systematic Review of the Literature[xii]

1. ***Purpose:*** *The broad goal of this review was to synthesize available data from studies evaluating the therapeutic efficacy of cannabis when used in combination with oncological treatments in cancer patients. Additionally, the study aimed to explore ongoing research in the field of oncology worldwide that investigates the use of cannabis in cancer treatment.*

2. ***Methods:*** *The study followed PRISMA guidelines and conducted a search in the MEDLINE/PubMed database between January 1, 2006, and March 1, 2022. Search terms included a range of cannabinoids and cannabis-related compounds (e.g., cannabidiol, THC, dronabinol, medical marijuana) and focused on studies examining the efficacy of cannabis administered during oncological treatments, irrespective of cancer type, localization, or sample size.*
3. ***Intervention:*** *The intervention studied was the use of cannabis in combination with oncological treatments in cancer patients. The review included studies where cannabis was administered to patients with glioblastoma and in combination with immunotherapy in various cancer subgroups. However, the results were insufficient to draw definitive conclusions about the therapeutic efficacy of cannabis in these settings.*
4. ***Conclusions:*** *The review concluded that, despite popular support for the idea that cannabis could be an ideal treatment for cancer, the current clinical trial data are insufficient to make definitive statements about its efficacy in combination with oncological treatments. There is a need for further clinical trials to clarify which combinations of chemotherapeutic agents and cannabinoids are most beneficial for cancer patients.*
5. ***Limitations:*** *Specific limitations include the insufficient data from clinical trials to draw conclusive results, as well as the fact that only a few studies have clearly examined the systemic effects of cannabinoids in cancer treatment. The review also highlights the need for more focused clinical trials to determine the most effective combinations of cannabinoids and conventional cancer therapies.*

Although the preceding review reported a lack of any consistent findings, clinical popularity is not necessarily indicative of medical efficacy, which was the subsequent systematic review's focus in investigating the biological effects of cannabinoids on human cancer cells. The findings demonstrated that cannabinoids effectively inhibited cancer cell growth, proliferation, and migration, while also exerting anti-inflammatory effects and reducing metastasis. These results suggested that cannabinoids possess potential anti-carcinogenic properties at the cellular level, providing a scientific basis for their therapeutic use in cancer treatment beyond merely palliative care.

Biological effects of cannabidiol on human cancer cells: Systematic review of the literature[xiii]

1. ***Purpose:*** *The broad goal of this systematic review was to examine the biological effects of cannabidiol (CBD), a major component of therapeutic cannabis, on various human pathological and cancer cell populations. The review specifically focused on cell viability, proliferation, migration, apoptosis, inflammation, metastasis, and CBD receptor expression across different body systems, including integumentary, gastrointestinal, genital and breast, respiratory, nervous, hematopoietic, and skeletal districts.*
2. ***Methods:*** *The review followed the PRISMA guidelines and involved an electronic search of databases such as MEDLINE via PubMed, Scopus, and Web of Science. The search was limited to English-language studies involving human cell lines and primary cultures from non-healthy donors, with CBD exposure as the variable and no CBD exposure as the control. Four independent reviewers performed the*

search, and 83 studies were selected from an initial pool of 3,974 articles. Quality assessment was conducted using the ToxRtool, with a reliability score ranging from 15 to 18.

3. ***Intervention:*** *The intervention examined was the exposure of human pathological and cancer cell populations to CBD. The results showed conflicting outcomes due to differences in CBD concentration, administration methods, and time points. CBD generally inhibited cell viability and proliferation in most cell types except for those in the integumentary system. The review also found significant inhibition of cell migration across all cell types and an increase in apoptosis at both high and low CBD doses. CBD exhibited anti-inflammatory effects on nervous cells at low doses and gastrointestinal cells at high doses, reduced metastatic potential at low doses, but increased angiogenesis in a skeletal cell line.*
4. ***Conclusions:*** *The review concluded that CBD has varying biological effects on different cell types, including the inhibition of cell viability, proliferation, migration, and metastasis, as well as the promotion of apoptosis and anti-inflammatory effects. These effects are mediated by specific receptors, such as CB1, CB2, and TRPV1, which are linked to viability, apoptosis, inflammation, and invasiveness. The review suggests that a detailed understanding of these effects could enable the therapeutic use of CBD while minimizing potential side effects.*
5. ***Limitations:*** *Specific limitations include the conflicting results across studies due to variations in CBD concentration, administration methods, and time points. These differences make it challenging to draw definitive conclusions about the overall effects of CBD. Additionally, the study was limited to in vitro research, which may not fully translate to clinical outcomes in human patients. The review also highlighted the need for further research to clarify the therapeutic potential of CBD and its receptor-mediated effects.*

Building on the cellular mechanisms observed in the preceding study, a systematic review focused on the use of cannabinoids in the treatment of melanoma explored various in vivo studies. The review found that cannabinoids demonstrated a capacity to inhibit tumor growth and induce apoptosis in cancer cells, highlighting their potential as a therapeutic option in melanoma. Despite these promising findings, the review noted significant limitations, including the heterogeneity of the studies examined and the absence of a clear consensus on the standard of care involving medical cannabis.

Roles of Cannabinoids in Melanoma: Evidence from In Vivo Studies[xiv]

1. ***Purpose:*** *The broad goal of this study was to review the existing in vivo evidence on the effects of cannabinoids, the major compounds of the Cannabis sativa L. plant, on melanoma, with the aim of exploring their potential as effective treatments with fewer side effects compared to existing therapies. The study specifically focused on the ability of cannabinoids to reduce tumor growth and induce apoptosis in melanoma cells.*

2. ***Methods:*** *The researchers conducted systematic searches across several databases, including PubMed, Embase, Scopus, and ProQuest Central, to identify relevant in vivo studies on the effects of cannabinoids on melanoma. The search included studies published from the inception of these databases. Out of 622 potential studies, six in vivo studies were deemed eligible and were included in the final analysis.*

3. ***Intervention:*** *The intervention studied involved the administration of cannabinoids, either individually or in combination, to assess their effects on melanoma cells. The overall results indicated that cannabinoids reduced tumor growth and promoted apoptosis and autophagy in melanoma cells.*

4. ***Conclusions:*** *The study concluded that cannabinoids have the potential to inhibit tumor growth and induce cell death in melanoma cells, highlighting their possible therapeutic benefits for treating melanoma. The review emphasized the need to better understand the mechanisms by which cannabinoids inhibit cancer-signaling pathways. Additionally, the study called for well-structured, randomized clinical trials in melanoma patients to validate cannabinoids as a viable and recognized therapeutic option for melanoma treatment.*

5. ***Limitations:*** *Specific limitations include the small number of eligible in vivo studies (only six), which limits the generalizability and robustness of the findings. The study also noted the need for further research to elucidate the underlying mechanisms of cannabinoid-mediated effects on melanoma, as well as the necessity for clinical trials to confirm their therapeutic potential in humans. Furthermore, the reliance on preclinical models may not fully capture the complexities of melanoma in human patients, necessitating caution in interpreting the results.*

Taken in aggregate, the seven systematic reviews and meta-analyses discussed in this literature review, which collectively encompass reviews of over a thousand studies, illustrate the inconsistent and often contradictory nature of current research on medical cannabis. These reviews present conflicting conclusions on key topics, including the presence and significance of health metrics, the efficacy of cannabis as an adjunct for managing cancer treatment symptoms, the overall viability of cannabis for cancer patients, and the nature and prevalence of adverse effects. The inconsistency in findings across these studies highlights the challenges in drawing definitive conclusions from the existing body of research. Addressing these issues requires more than isolated systematic reviews; it necessitates a comprehensive and inclusive meta-analysis approach that leverages big data to provide a more robust and reliable assessment of cannabis's medical potential.

CBD vs. full extract cannabis

Due to legal issues and a desire to avoid the psychoactive effects of THC, CBD has become a popular substitute for studies exploring medical cannabis.[xv] However, studies focusing solely on CBD are not necessarily indicative of the efficacy of full extract cannabis, which includes THC, CBD, and a range of other cannabinoids and terpenes. This distinction is crucial due to the complex pharmacodynamics involved in cannabis use, where the combination of different cannabinoids and terpenes can lead to synergistic or antagonistic effects, commonly referred to as the "entourage effect."[xvi]

CBD and THC interact with the endocannabinoid system in different ways, primarily through CB1 and CB2 receptors, but their combined effects can modify the pharmacological profile of cannabis significantly.[xvii] For instance, while THC is a partial agonist at CB1 receptors and is primarily responsible for the psychoactive effects of cannabis, CBD acts as a negative allosteric modulator at these receptors, which can mitigate the psychoactive effects of THC.[xviii] This interaction can potentially enhance the therapeutic window of cannabis, allowing for effective symptom management with reduced adverse effects, such as anxiety or psychoactive impairment that might occur with THC alone.

Moreover, terpenes and other minor cannabinoids present in full extract cannabis can further influence the therapeutic efficacy by modulating the pharmacokinetics and pharmacodynamics of both THC and CBD.[xix] For example, certain terpenes may enhance the permeability of cell membranes, facilitating the uptake of cannabinoids, or may affect the metabolism of cannabinoids by interacting with cytochrome P450 enzymes, thereby altering their bioavailability and duration of action.[xx]

These complex interactions suggest that the effects observed in studies focusing solely on CBD may not fully represent the potential therapeutic outcomes of using full extract cannabis. The presence of THC and other cannabinoids could either potentiate or diminish the therapeutic effects of CBD, depending on the condition being treated and the specific cannabinoid and terpene profile of the cannabis extract. Therefore, conclusions drawn from CBD-only studies should be interpreted with caution, as they may not fully capture the broader therapeutic potential or risks of full spectrum cannabis extracts.

Methodology

Definitions

It is necessary to define key terms to ensure clarity and precision in understanding the research approaches and findings. Below are fundamental terms and their contextual use in this study:

1) **Correlations**: In the context of this meta-analysis, correlations refer to statistical measures that describe the strength and direction of a linear relationship between two variables. These are quantified using Pearson's correlation coefficient, *r*, to determine how strongly associated the presence of specific keywords or topics is with various outcomes in the cannabis studies.

2) **Sentiment Analysis**: Sentiment analysis in this research refers to the computational process of identifying and categorizing opinions expressed in the text data from research articles, specifically to determine the tone and implications of the studies concerning cannabis. This analysis differentiates between positive (Supported), negative (Not Supported), and indeterminate (Unclear) sentiments reflected in the literature.

a) **Supported Sentiment**: Supported sentiment indicates a prevalence of terms associated with positive outcomes, the evidence supporting the efficacy or beneficial impact of cannabis, or otherwise determining the benefits outweigh the risks in a therapeutic context.

b) **Not Supported Sentiment**: Not supported sentiment indicates a prevalence of terms associated with negative outcomes, the evidence disputing the efficacy or beneficial impact of cannabis, or otherwise determining the risks outweigh the benefits in a therapeutic context.

c) **Unclear Sentiment**: Unclear sentiment is used to describe studies that do not have a clear prevalence of Supported or Not Supported terms, resulting in an indeterminate sentiment that neither supports nor rejects cannabis' efficacy, but indicates the need for more information.

3) **Keyword Occurrences**: This term refers to the frequency with which specific words or phrases (keywords) appear in the cannabis-related studies analyzed. Keyword occurrences are used to both determine initial sentiment analysis and the correlation of topics with those sentiments. In this meta-analysis, keyword occurrences are used to identify and quantify the presence of relevant terms within the literature and their association with research outcomes, providing insights into the patterns within research trends.

4) **Dominant Instances**: Dominant instances define the number of studies that were categorized as a whole to be in the Supported, Not Supported, or Unclear categories, based on the predominant sentiment-associated keywords in an article. This concept is used to gauge the prevailing or most emphasized sentiment within individual studies, providing a weighted measure of their significance in the broader analysis. Unlike keyword occurrences, dominant instances use a zero-sum categorization, if an article presents more keywords associated with Not Supported sentiment, the article is categorized as Not Supported and any Supported or Unclear keywords within that article are not counted in the analysis.

5) **Sensitivity Analysis**: In this study, sensitivity analysis involves testing how the results of the meta-analysis vary with changes in the methodology, such as the recalibration of keyword weights or the redefinition of sentiment categories. This process helps in verifying the robustness and reliability of the findings, ensuring they are not unduly influenced by methodological biases or anomalies. This was performed by recalculating sentiment analysis through the lens of Dominant Instance-based sentiments. This method aims to neutralize the impact of outliers and mitigate the influence of banal term confounders, providing a more balanced and accurate reflection of the sentiment landscape.

6) **Therapeutic Cannabis**: This term refers to the use of cannabis or its derivatives primarily for the purpose of health benefits, quality of life improvement, symptom management, or disease treatment. Therapeutic cannabis refers to the qualified, controlled use of cannabinoids, not to recreational use or abuse. In this meta-analysis, therapeutic cannabis encompasses studies examining its efficacy and safety across various medical conditions and patient outcomes.

Search terms

Search Term	Results
("cannabis"[MeSH Terms] OR "cannabinoids"[MeSH Terms] OR "cannabis"[All Fields]) AND ("receptor"[Title/Abstract] OR "endocannabinoid system"[All Fields])	6400
(cannabis[Title/Abstract]) AND (symptoms[Title/Abstract])	3949
("cannabis"[MeSH Terms] OR "cannabis"[All Fields]) AND ("endocrine system"[MeSH Terms] OR "hormones"[All Fields] OR "cytokines"[MeSH Terms] OR "immune system"[MeSH Terms] OR "immunology"[All Fields])	3711
("cannabis"[MeSH Terms] OR "cannabis"[All Fields]) AND ("mood"[MeSH Terms] OR "mental health"[All Fields])	3666
(cannabis[Title/Abstract]) AND (abuse[Title/Abstract])	3526
("cannabis"[MeSH Terms] OR "cannabis"[All Fields]) AND ("endocrine system"[MeSH Terms] OR "hormones"[All Fields])	3262
("cannabis"[MeSH Terms] OR "cannabis"[All Fields]) AND ("pain"[MeSH Terms] OR "pain"[All Fields])	2790
(cannabis[Title/Abstract]) AND (therapeutic[Title/Abstract])	2519
("cannabis"[All Fields] OR "cannabinoids"[All Fields]) AND ("neoplasms"[MeSH Terms] OR "cancer"[All Fields] OR "oncology"[All Fields])	2511
(cannabis[Title/Abstract]) AND (adverse[Title/Abstract])	2116
("cannabinoids"[MeSH Terms] OR "cannabinoids"[All Fields]) AND ("neoplasms"[MeSH Terms] OR "cancer"[All Fields] OR "oncology"[All Fields])	1805
(cannabis[Title/Abstract]) AND (addiction[Title/Abstract])	1730
("cannabis"[MeSH Terms] OR "cannabis"[All Fields]) AND ("neoplasms"[MeSH Terms] OR "cancer"[All Fields] OR "oncology"[All Fields])	1689
("cannabis"[All Fields] OR "cannabinoids"[All Fields]) AND ("cardiovascular system"[MeSH Terms] OR "heart"[All Fields] OR "blood vessels"[All Fields] OR "neurology"[MeSH Terms] OR "neurological system"[All Fields] OR "physiology"[MeSH Terms] OR "physiological processes"[All Fields] OR "neurotransmitters"[MeSH Terms] OR "neurotransmitters"[All Fields])	1588
(medical cannabis[Title/Abstract])	1539
("cannabinoids"[MeSH Terms] OR "cannabinoids"[All Fields]) AND ("CB1 receptor"[MeSH Terms] OR "CB1 receptor"[All Fields])	1493

("cannabis"[MeSH Terms] OR "cannabis"[All Fields]) AND ("endocannabinoid system"[MeSH Terms] OR "endocannabinoid system"[All Fields])	1355
("cannabinoids"[MeSH Terms] OR "cannabis"[All Fields] OR "CBD oil"[All Fields]) AND ("inflammation"[MeSH Terms] OR "inflammation"[All Fields])	1344
("cannabinoids"[MeSH Terms] OR "cannabinoids"[All Fields]) AND ("inflammation"[MeSH Terms] OR "inflammation"[All Fields])	1324
((inflammat[Title/Abstract]) OR (inflammation[MeSH Major Topic])) AND (cbd[Title/Abstract])	978
(medical marijuana[Title/Abstract])	948
("cannabinoids"[MeSH Terms] OR "cannabinoids"[All Fields]) AND ("cytokines"[MeSH Terms] OR "cytokines"[All Fields])	784
("cannabis"[MeSH Terms] OR "cannabis"[All Fields]) AND ("inflammation"[MeSH Terms] OR "inflammation"[All Fields])	753
("cannabinoids"[MeSH Terms] OR "cannabinoids"[All Fields]) AND ("apoptosis"[MeSH Terms] OR "apoptosis"[All Fields] OR "cell cycle"[All Fields] OR "angiogenesis"[All Fields] OR oncology[Title/Abstract])	751
"CBD"[Title/Abstract] OR "cannabidiol"[All Fields]) AND ("inflammation"[MeSH Terms] OR "inflammation"[All Fields])	718
("cannabis"[MeSH Terms] OR "cannabis"[All Fields]) AND ("cardiovascular system"[MeSH Terms] OR "heart"[All Fields] OR "blood vessels"[All Fields])	699
("cannabis"[MeSH Terms] OR "cannabis"[All Fields]) AND ("inflammation"[MeSH Terms] OR "inflammation"[All Fields])	690
("cannabinoids"[MeSH Terms] OR "cannabinoids"[All Fields]) AND ("CB2 receptor"[MeSH Terms] OR "CB2 receptor"[All Fields])	654
(cannabis[Title/Abstract]) AND (inflammation[Title/Abstract])	615
("cannabinoids"[MeSH Terms] OR "cannabinoids"[All Fields]) AND ("apoptosis"[MeSH Terms] OR "apoptosis"[All Fields])	570
("cannabis"[MeSH Terms] OR "cannabis"[All Fields]) AND ("immune system"[MeSH Terms] OR "immunology"[All Fields])	486
("cannabis"[MeSH Terms] OR "cannabis"[All Fields]) AND ("appetite"[MeSH Terms] OR "appetite"[All Fields])	397
("cannabinoids"[MeSH Terms] OR "cannabinoids"[All Fields]) AND ("biomarkers"[MeSH Terms] OR "biomarkers"[All Fields])	370
("cannabis"[MeSH Terms] OR "cannabis"[All Fields]) AND ("biomarkers"[MeSH Terms] OR "biomarkers"[All Fields])	353
("cannabinoids"[MeSH Terms] OR "cannabinoids"[All Fields]) AND ("neurotransmitters"[MeSH Terms] OR "neurotransmitters"[All Fields])	229

("cannabinoids"[MeSH Terms] OR "cannabinoids"[All Fields]) AND ("cell cycle"[MeSH Terms] OR "cell cycle"[All Fields])	201
(cannabis[Title/Abstract]) AND (cytokines[Title/Abstract])	135
("cannabis"[MeSH Terms] OR "cannabis"[All Fields]) AND ("neurotransmission"[MeSH Terms] OR "neurotransmission"[All Fields])	135
("cannabis"[MeSH Terms] OR "cannabis"[All Fields]) AND ("oncology"[MeSH Terms] OR "cancer"[All Fields] OR "tumor"[All Fields]) AND ("mechanism"[All Fields])	97
("cannabis"[MeSH Terms] OR "cannabis"[All Fields]) AND ("physiology"[MeSH Terms] OR "physiological processes"[All Fields])	72
("cannabis"[MeSH Terms] OR "cannabis"[All Fields]) AND ("angiogenesis inhibitors"[MeSH Terms] OR "angiogenesis"[All Fields])	52
(full extract cannabis oil[All Fields])	19
((full extract cannabis oil) OR (full spectrum cannabis oil)) AND ("CBD"[Title/Abstract] OR "cannabidiol"[All Fields])	15
("full spectrum cannabis oil"[MeSH Terms] OR "full spectrum cannabis oil"[All Fields])	14
((full extract cannabis oil) OR (full spectrum cannabis oil)) AND ("THC"[Title/Abstract] OR "tetrahydrocannabinol"[All Fields])	13
("cannabis"[MeSH Terms] OR "cannabis"[All Fields]) AND ("neurology"[MeSH Terms] OR "neurological system"[All Fields])	6
**Initial Total**	**59071**
**Duplicates/Text Unavailable/Unreadable**	**48430**
**Final Total**	**10641**

Table 1 List of search terms entered into PubMed with a filter active for "available abstract," using specific keywords and Medical Subject Headings (MeSH), then filtered for duplicate entries, missing text, or unreadable content.

Keyword Selection

Keyword selection is foundational for conducting sentiment analysis within the context of a meta-analysis, especially when examining the large and heterogeneous body of research on medical cannabis. The objective of sentiment analysis in this scenario is to categorize studies based on the language they use; whether they support the use of medical cannabis, do not support its use, or present unclear conclusions. Given the diversity in how conclusions are expressed across studies, a broad array of keywords must be identified to accurately reflect the sentiment conveyed in each study.

Beyond the basic sentiment categorization, a deeper correlational analysis necessitates the identification of keywords related to potential factors influencing study outcomes. This step involves compiling collections of keywords that correspond to a variety of factors relevant to medical cannabis research. These factors include, but are not limited to, the types of cannabis used (e.g., THC vs. CBD dominant strains), biological markers such as cytokines, and clinical outcomes like tumor growth, shrinkage, or size. Additionally, keywords related to specific cancers, treatment effects (e.g., remission, inflammation, anti-inflammatory effects), symptom management (appetite, nausea, anxiety, depression, quality of life, pain), and the broader therapeutic context (endocannabinoids, opioids, opioid addiction, addiction) are gathered. The analysis also considers treatment modalities (immunotherapy, radiation therapy, chemotherapy) and outcomes (anti-carcinogenic effects, survival rates) pertinent to cancer treatment.

Achieving parity in the allocation of keywords for sentiment analysis is vital to ensure unbiased interpretation of evidence. With 97 keywords identified for supporting evidence and 102 keywords for not supporting evidence, the distribution is relatively balanced, with supporting keywords constituting approximately 48.7% and not supporting keywords about 51.3% of the total keywords allocated for these sentiments (199 keywords in total). This close percentage distribution ensures that the sentiment analysis does not inherently favor one viewpoint over the other due to an imbalance in keyword quantity.

The allocation for keywords suggesting unclear evidence stands at 55, which is significantly lower compared to the other two categories. This distribution reflects most studies' wide-ranging ways of distinguishing between positions of support or opposition of evidence, in comparison to well-established expressions for ambiguity. The proportion of unclear keywords is approximately 21.6% when considering the total count of keywords across all three sentiment categories (254 keywords in total).

Correlations

Correlations, also referred to as associations, are employed as the primary metric to elucidate patterns and associations within the extensive dataset of medical cannabis research. Correlation coefficients, such as Pearson's *r*, are statistical measures that quantify the degree to which two variables are related. While correlation does not explain causation, correlation patterns can nonetheless help predict outcomes.

Relevance of Pearson's r

The application of Pearson's correlation coefficient, denoted as *r*, in the context of a systematic review meta-analysis involving medical cannabis research, plays a pivotal role in identifying relationships between different variables within a dataset. Specifically, *r* is used to measure the strength and direction of the linear relationship between two variables—here, the occurrence rates of keywords within supported, not supported, and unclear sentiment categories and each topic group of keywords.

Pearson's correlation coefficient is highly relevant in analyzing large datasets, especially those exceeding 1 million data points, as it provides a quantifiable measure of the degree to which two variables are linearly related. This capability is particularly valuable in systematic review meta-

analyses where the sheer volume of data precludes manual inspection of relationships between variables. In the context of medical cannabis research, *r* enables researchers to systematically assess how sentiment toward medical cannabis (supported, not supported, unclear) correlates with specific areas of research focus, as denoted by keyword occurrences, which is crucial for:

- **Identifying Consensus and Controversy**: By correlating sentiment categories with research topics, Pearson's *r* can highlight areas of research where there is a strong consensus or significant controversy regarding the efficacy or safety of medical cannabis.
- **Detecting Subtle Trends**: Correlations between keyword occurrences and sentiments may reveal new trends in medical cannabis research, indicating convergence of scientific inquiry and topics that are not readily apparent without pattern analysis.
- **Guiding Future Research**: The identification of topics with weak or negative correlations with supportive evidence can signal gaps in the current literature, guiding future research efforts toward these under-explored areas.

Pearson's *r* ranges from -1 to +1, where +1 indicates a perfect positive linear relationship, -1 indicates a perfect negative linear relationship, and 0 signifies no linear relationship.[xxi] This range provides a straightforward interpretation of the relationship dynamics between different sets of keywords and sentiments, offering clear insights into the alignment or divergence within the body of literature. In accordance with the large dataset and breadth of potential associations, a more refined set of correlation strengths has been implemented.[xxii]

In ensuring technical accuracy, it is essential to adhere to established statistical principles when calculating and interpreting Pearson's *r*. This includes ensuring data normality, linearity between variables, and the absence of outliers that could skew the results. For large datasets, the significance of the calculated *r* values should also be tested to determine whether the observed correlations are statistically significant and not due to random chance. [xxiii]

T-scores and *p* -values

In the methodology for analyzing correlations within a systematic review and meta-analysis, calculating t scores and deriving *p* values from these scores are essential steps for determining the statistical significance of the correlations observed between keywords related to medical cannabis research topics and sentiments.

The calculation of t scores in the context of correlation analysis follows after obtaining Pearson's correlation coefficient (*r*) between two variables. The t score is computed using the formula:

$$ t = r\sqrt{\frac{n-2}{1-r^2}} $$

where *r* is the Pearson correlation coefficient and *n* is the number of data points, or studies, included in the analysis. This formula translates the correlation coefficient into a t score under the assumption that the underlying relationship between the variables is linear, and the data are normally distributed.

Following the calculation of the t score, *p* values are derived to assess the statistical significance of the correlation. The *p* value represents the probability of observing a correlation as strong as the one detected (or stronger) if there were actually no such correlation in the population from which the sample was drawn. The adopted threshold for significance was $p < 0.05$, indicating that there is less than a 5% chance that the observed correlation occurred by random chance.

Given the high volume of comparisons and the risk of Type I errors (falsely identifying a meaningful effect), applying a significance threshold helps in prioritizing findings that are statistically reliable.

Sentiment analysis keywords

**Supported**	**Not Supported**	**Unclear**
advancing research	adverse	ambiguous
advantageous	adverse findings	anecdotal
alleviate	advised against	deficient evidence
ameliorate	aggravate	dubious
assuage	agitate	equivocal
assuring	amplify	erratic
auspicious	antagonistic	evidence-deficient
beneficial	baleful	fallible
beneficial outcomes	cannabis abuse	fluctuating
better	cannabis dependency	heterogeneous
cannabis demonstrates efficacy	cannabis intoxication disorder	hypothetical
cannabis is advantageous	cannabis use disorder	inaccurate
cannabis is beneficial	chronic marijuana use	inadequate evidence
cannabis is effective in treatment	compound	incomplete evidence
cannabis is efficacious	compromise	inconclusive
cannabis is helpful	counterproductive	inconclusive evidence
cannabis is potent	damaging	inconsistent
cannabis is therapeutic	decline	indeterminate
cannabis provides relief	degenerate	insufficient data

cannabis shows positive effects	degrade	insufficient evidence
capable	deleterious	irrelevant
competent	depreciate	lacking evidence
constructive	destabilize	limited evidence
constructive results	destructive	minimal evidence
curative	deteriorate	non-conclusive
effective results	deteriorate	nondefinitive
efficacious	detrimental	non-validated
efficient	detrimental effect	not definitive
elevate	devaluate	not enough evidence
encouraging	disadvantageous	open-ended
enhance	discouraged	premature
enhancing regulatory functions	encouraging	provisional
favorable	escalate	questionable
functional	exacerbate	scarce evidence
healing	forbidden	sparse evidence
healthful	futile	speculative
heartening	harmful	tentative
helpful	hostile	uncertain
hopeful	ill effects	unclear
impactful	impair	unconfirmed
improve	inadequate	unconvincing
improved outcomes	inadvisable	unconvincing evidence
medicinal	inappropriate for use	undependable
mitigate	inconclusive	undetermined
nutritious	inconsequential	unestablished evidence
operative	ineffective	unfounded
optimistic	ineffectual	unknown
optimize	inefficacious	unproven
palliate	injurious	unresolved

palliative	inoperative	unsubstantial evidence
positive	insufficient	unsubstantiated
positive implications	insufficient evidence	unsupported
potent	insufficient evidence	untrustworthy
potential	intensify	unverified
preventative	lacking support	weak evidence
preventive	limited efficacy	
productive	low efficacy	
proficient	maladaptive	
progressing studies	maleficent	
promising	malicious	
promising evidence	marijuana addiction	
promotive of regulation	marijuana dependency	
prophylactic	marijuana misuse	
pro-regulation	minimal impact	
prospective	negative	
protective	negative implications	
rectify	negative outcomes	
refine	non-beneficial	
regulation	non-effective	
regulation augmenting	non-efficacious	
regulation boosting	nonresponsive	
regulation encouraging	non-supportive	
regulatory	not indicated	
regulatory advancing	not recommended	
regulatory enhancing	not suitable	
regulatory facilitating	not to be used	
regulatory positive	noxious	
regulatory promoting	null	
regulatory stimulatory	pernicious	

rehabilitative	problematic marijuana use	
relieve	prohibited	
remedial	recreational cannabis abuse	
remediate	side effects	
restorative	suboptimal	
result-oriented	suboptimal results	
salutary	subvert	
significant efficacy	to be avoided	
stimulatory on regulation	toxic	
substantiated	undermine	
successful	undesirable	
supportive	unfavorable	
supportive findings	unhealthy	
supportive of regulatory mechanisms	unproductive	
therapeutic	unpromising results	
up-and-coming	unpropitious	
upgrade	unsettle	
uplifting	unsuccessful	
	unwelcome	
	upset	
	useless	
	void	
	weaken	

Table 2a Keywords for sentiment analysis

Topic keywords

**Tumor Growth**	**Tumor shrink**	**Tumor size**	**Cancers**	**Cancerous**
Tumor size increase	tumor shrink	Tumor size	Cancer	Malignant
Neoplasm proliferation	Tumor shrinkage	Lesion size	Cancers	Cancerous

Tumoral expansion	Neoplasm diminution	Neoplasm diameter	Carcinoma	Neoplastic
Malignancy progression	Tumoral decrease	Growth dimension	Malignancy	Oncogenic
Cancerous growth	Malignancy reduction	Mass extent	Neoplasm	Carcinogenic
Neoplastic growth	Cancer shrinkage	Carcinoma extent	Tumor	Tumorigenic
Tumor enlargement	Lesion reduction	Tumoral extent	Oncology	Metastatic
Tumor progression	Tumor regression	Neoplastic size	Sarcoma	Dysplastic
Oncogenesis	Neoplastic regression	Malignancy size	Lymphoma	Pre-cancerous
Carcinogenesis	Tumor size decrease	Tumor volume	Leukemia	Carcinomatous
Tumorigenesis	Reduction in tumor volume	Tumoral volume	Metastasis	Sarcomatous
Neoplastic enlargement	Tumoral response		Adenocarcinoma	
Tumor development	Oncologic response		Basal cell carcinoma	
Tumor amplification	Partial response		Squamous cell carcinoma	
Tumor increase	Tumor debulking		Melanoma	
Growth of tumor			Glioma	
			Myeloma	
			Carcinogenesis	

			Oncogenesis	
			Neoplastic	

Table 3a Keyword allocations for cancer dynamics, Part I of II

**Survival**	**Therapeutic**	**Cytokines**	**Anti inflammatory**	**Inflammation**
DFS	Clinical	C-C motif chemokine ligand	Anti-inflammation	Acute inflammation
Disease-free survival	Curative	CCL	Anti-inflammation agents	Anti-inflammatory (for agents that reduce inflammation)
Five-year survival rate	Healing	Chemokines	Anti-inflammatory	ASC complex
Life expectancy	Health-promoting	Colony-Stimulating Factors	Anti-inflammatory agents	Autoimmune-related inflammation
Long-term survival	Interventional	CSF	Corticosteroids	CARD complex
Median survival	Medicinal	C-X-C motif chemokine ligand	Immunomodulatory	Caspase activation and recruitment domain complex
Mortality rate	Palliative	CXCL	Inflammation control	Chronic inflammation
Overall survival	Pharmacotherapeutic	Cytokine	Inflammation dampening	Chronic inflammatory condition
Patient survival	Preventative	Cytokines	Inflammation modulation	Chronic inflammatory disease
PFS	Preventive	EPO	Inflammation-attenuating	Chronic inflammatory process
Progression-free survival	Rehabilitative	Erythropoietin	Inflammation-blocking	Continuous inflammation
Relapse-free survival	Remedial	FGF	Inflammation-inhibiting	Cytokine-inducing
Survival analysis	Restorative	Fibroblast Growth Factors	Inflammation-reducing	Cytosolic multiprotein oligomer
Survival outcome	Therapeutic	G-CSF	Inflammatory suppression	Enduring inflammation
Survival percentage	Therapeutical	GM-CSF		Immune-activating
Survival probability	Treatment-related	Granulocyte Colony-Stimulating Factor		Infection-induced inflammation
Survival rate		Granulocyte-Macrophage Colony-Stimulating Factor		Inflamed
Survival statistics		Growth Factors		Inflammasome assembly
		IFN		Inflammation
		IFN-gamma		Inflammation, chronic
		IL-1		Inflammation-associated complex
		IL-10		Inflammation-enhancing
		IL-2		Inflammation-inducing
		IL-6		Inflammation-related

		Interferon-gamma		Inflammation-stimulating
		Interferons		Inflammatory caspase activating complex
		Interleukin-1		Inflammatory condition
		Interleukin-10		Inflammatory mediator-releasing
		Interleukin-2		Inflammatory process
		Interleukin-6		Inflammatory response
		Interleukins		Inflammatory response complex
		PDGF		Inflammatory response-triggering
		Platelet-Derived Growth Factors		Inflammatory-promoting
		TGF-beta		Inflammogenic
		TNF		Innate immune system complex
		TNF-alpha		Irritated
		Transforming Growth Factor-beta		Long-standing inflammation
		Tumor Necrosis Factor-alpha		Long-term inflammation
		Tumor Necrosis Factors		NLR complex
				NLRP3 inflammasome
				NOD-like receptor complex
				Non-resolving inflammation
				Persistent inflammation
				Pro-inflammatory
				Proinflammatory cytokine-producing
				Protracted inflammation
				Pyrin domain-containing protein complex
				Reddened
				Sustained inflammatory response
				Swollen

Table 4a Keyword allocations for health metrics and markers

**cannabis types**	**Endocannabinoids**
broad-spectrum cbd oil	2-Arachidonoylglycerol (2-AG)
cannabichromene	2-Arachidonyl glyceryl ether (noladin ether)

cannabidiol	Anandamide (AEA)
cannabidiol oil	Cannabinoid receptor ligands
cannabigerol	Eicosanoids
cannabinoid therapy	Endocannabinoid
cannabinoid-based therapy	Endocannabinoids
cannabinoids	N-Arachidonoylethanolamine (AEA)
cannabinol	Oleoylethanolamide (OEA)
cannabis concentrate	Palmitoylethanolamide (PEA)
cannabis extract oil	Virodhamine (OAE)
cannabis indica	
cannabis ruderalis	
cannabis sativa	
cannabis therapy	
cannabis-based medicines	
cbc	
cbd	
cbd extract	
cbd hemp oil	
cbd therapy	
cbd tincture	
cbd-rich oil	
cbg	
cbn	
concentrated cannabis tincture	
delta-9-tetrahydrocannabinol	
endocannabinoids	
feco	
full-spectrum cbd oil	
hemp extract oil	
hemp oil	
high-potency cannabis oil	
medical cannabis oil	
medical cannabis treatment	
medical cannabis use	
medicinal cannabis	
phytocannabinoid-rich (pcr) oil	

phytocannabinoids	
pure cannabis oil	
rick simpson oil (rso)	
tetrahydrocannabinol	
tetrahydrocannabivarin	
thc	
thc therapy	
thcv	
therapeutic cannabis	
whole extract cannabis oil	
whole plant extract	

Table 5a Keyword allocation for cannabinoid types

**Remission**	**Immunotherapy**	**Radiation Therapy**	**chemotherapy**	**Anti carcino**
Remission	Immunotherapy	Radiotherapy	chemo	Antineoplastic
Oncologic remission	Biological therapy	Radiation oncology	chemotherapy	Anticarcinogenic
Tumor regression	Biotherapy	External beam radiation therapy (EBRT)	antineoplastic therapy	Antitumoral
Disease remission	Cancer immunotherapy	Internal radiation therapy	cytotoxic therapy	Antitumor
Neoplastic remission	Immuno-oncology	Brachytherapy	cancer pharmacotherapy	Cancer-fighting
Cancer suppression	Monoclonal antibodies therapy	Stereotactic radiotherapy	oncologic chemotherapy	Chemopreventive
Malignancy remission	Checkpoint inhibitor therapy	Stereotactic radiosurgery (SRS)	chemotherapeutic treatment	Oncolytic
Complete remission	Adoptive cell transfer	Intensity-modulated radiation therapy (IMRT)	anticancer therapy	Tumor-suppressive
Partial remission	Cancer vaccines	Image-guided radiation therapy (IGRT)	adjuvant chemotherapy	Carcinostatic
Clinical remission	Cytokine therapy	Proton therapy	neoadjuvant chemotherapy	Neoplasm-inhibitory

Pathological remission	Immune checkpoint therapy	Particle therapy	systemic therapy	Cancer-inhibitory
Hematologic remission	CAR T-cell therapy	Gamma knife radiotherapy	intravenous chemotherapy	Tumoricidal
Tumoral remission	Tumor-infiltrating lymphocytes therapy	Cyberknife radiotherapy	oral chemotherapy	Chemotherapeutic
Remission induction	Interleukin therapy	Adjuvant radiation therapy	palliative chemotherapy	Carcinoma-inhibitive
Response to treatment	Interferon therapy	Neoadjuvant radiation therapy	targeted chemotherapy	Oncostatic
	Immune modulation therapy	Palliative radiation therapy		
	Dendritic cell therapy	radiation therapy		

Table 6a Keyword allocations for cancer dynamics, Part II of II

**Appetite**	**Nausea**	**Anxiety**	**Depression**
Hunger	Queasiness	Generalized anxiety disorder	Depression
Appetite	Stomach upset	GAD	Major depressive disorder
Craving	Sickness	Anxiety	MDD
Desire for food	Emesis	Panic disorder	Clinical depression
Nutritional intake desire	Nauseous feeling	Social anxiety disorder	Unipolar depression
Satiety	Gastric distress	SAD	Dysthymia
Feeding desire	Motion sickness	Phobias	Seasonal affective disorder
Anorexia	Morning sickness	Anxiety disorders	SAD
Hyperphagia	Chemotherapy-induced nausea	Obsessive-compulsive disorder	Depressive episode

Polyphagia	Postoperative nausea	OCD	Depressive mood
Eating drive	Nauseous	Post-traumatic stress disorder	Melancholia
Food intake regulation	N&V	PTSD	Mood disorder
Appetitive behavior	Seasickness	Anxiety syndrome	Affective disorder
Hunger sensation	Gastrointestinal discomfort	Stress-related anxiety	Depressive illness
Caloric intake desire	Anticipatory nausea	Acute stress reaction	Endogenous depression
		Chronic anxiety	Exogenous depression
		Anxiety symptoms	Reactive depression
		Neurotic anxiety	Neurotic depression
		Psychogenic anxiety	Atypical depression
		Anxiolytic	

Table 7a Keyword allocations for conditions Part I of II

**Quality of life**	**Pain**	**Opioids**	**Opioid Addiction**	**Addiction**
Quality of life	Pain	Opioids	Opioid addiction	Addiction
Life quality	Analgesia	Opiates	Opioid use disorder (OUD)	Substance use disorder
Health-related quality of life	Pain management	Morphine	Opioid dependence	Dependency
HRQoL	Pain control	Codeine	Opioid abuse	Chemical dependency
Well-being	Pain reduction	Heroin	Opiate addiction	Habituation
Patient-reported outcomes	Pain alleviation	diacetylmorphine	Opioid substance abuse disorder	Psychological dependence
PROs	Pain suppression	Synthetic opioids	Narcotic addiction	Physical dependence
Living standards	Anesthetic effect	Fentanyl	Opioid dependency syndrome	Drug addiction

Life satisfaction	Pain mitigation	Oxycodone	Prescription opioid addiction	Alcohol addiction
Wellness	Pain remedy	Hydrocodone	Opioid misuse disorder	Nicotine addiction
Functional status	Pain abatement	Methadone	Chronic opioid abuse	Opioid addiction
Physical and mental health composite scores	Pain therapy	Buprenorphine	Opiate dependence syndrome	Compulsive behavior
Subjective well-being	Pain treatment	Tramadol	Opioid-related disorder	Drug abuse
Health status	Antinociception	Opioid analgesics	Opioid-induced disorder	Substance abuse
Social functioning	Pain modulation	Narcotic analgesics		Chronic relapse
Psychological well-being	Pain easing	Semi-synthetic opioids		Withdrawal syndrome
Life condition	Palliative care	Opioid receptors		Tolerance
Standard of living	Symptom management	Opioid agonists		
	Supportive care	Opioid antagonists		
	Comfort care	naloxone		
	End-of-life care	Opioid peptides		
	Hospice care			
	end-of-life			
	Symptom control			
	Palliative treatment			
	Palliative therapy			
	Quality of life care			
	Non-curative treatment			
	Relief care			
	Palliative intervention			

Table 8a Keyword allocations for conditions Part II of II

Keyword Occurrences

The methodology for tracking the occurrence of keywords within individual studies in the systematic review dataset involves a detailed, quantitative approach to textual analysis. This method is instrumental in uncovering patterns and themes across a large volume of literature on medical cannabis, facilitating a nuanced understanding of the research landscape. Here's an outline of this process:

1. **Keyword Tracking**: For each study in the dataset, the frequency of specified keywords related to medical cannabis is carefully recorded. These keywords are organized by topics, such as types of cannabis, therapeutic effects, specific medical conditions (e.g., various cancers), treatment-related terms (e.g., chemotherapy, immunotherapy), and outcomes (e.g., tumor growth, quality of life). The tracking process involves a systematic scan of each study's text—often focusing on the abstract, results, and discussion sections—to tally the occurrences of each keyword.

2. **Topic Allocation**: Keywords are categorized into broader topics to streamline the analysis. For example, "tumor increase," "tumor growth," and "tumor size" might fall under a "Tumor Growth" topic, while "generalized anxiety" and "chronic stress" might be grouped under the "Anxiety" topic. This categorization helps in organizing the data and facilitates a topic-wise analysis of keyword occurrences.

3. **Total Occurrences per Topic**: The total number of keyword occurrences is calculated for each topic within each study. This aggregate figure provides insight into which aspects of medical cannabis are most frequently discussed or emphasized in the literature, highlighting areas of significant interest or concern within the research community.

4. **Categories, Topics, Keywords**: The structure of categories, topics, and individual keywords is utilized to organize and analyze the vast amount of keywords captured in this meta-analysis.

   4.1. ***Category***: Categories are broad areas that encompass specific areas of interest within the field.

      4.1.1. ***Topic***: Each category is further divided into topics, which are more focused areas of study within the general category.

         4.1.1.1. ***Keyword***: These topics are then made up of individual keywords, which are the specific terms or phrases extracted from the studies themselves.

Categories

Health metrics

- **(Anti-) Inflammation**: Inflammation plays a critical role in the pathogenesis and progression of cancer. Chronic inflammation can promote tumor growth by creating a microenvironment conducive to cancer cell proliferation, survival, and metastasis. In the context of cancer, measuring inflammatory markers helps to assess disease progression, treatment response, and overall prognosis. Therapeutic strategies that focus on anti-inflammatory effects may potentially slow tumor progression and improve patient outcomes.

- **Therapeutics**: Therapeutics encompass all treatments aimed at managing cancer, including pharmaceutical drugs, biologics, and integrative therapies. The relevance of therapeutics within health metrics lies in their ability to influence patient outcomes by modulating physiological processes such as cell proliferation, apoptosis, and immune response. In cancer treatment, evaluating therapeutic efficacy is essential for determining the success of interventions, managing side effects, and improving quality of life.

Cancer Treatments

- **Appetite**: Appetite regulation is a significant concern in cancer treatment, particularly in patients undergoing chemotherapy or suffering from advanced disease. Cancer-related cachexia and anorexia are associated with severe weight loss and malnutrition, which can negatively impact treatment outcomes and overall survival. Treatments that improve appetite can help maintain nutritional status and enhance patients' ability to tolerate aggressive cancer therapies.
- **Chemotherapy**: Chemotherapy is a cornerstone of cancer treatment, involving the use of cytotoxic drugs to kill or inhibit the growth of cancer cells. Its relevance in this category lies in its widespread application across various cancer types and its significant impact on both tumor control and patient quality of life. Evaluating chemotherapy's effectiveness and managing its side effects, such as nausea and pain, are critical aspects of comprehensive cancer care.
- **Nausea**: Nausea is one of the most common and debilitating side effects of chemotherapy and other cancer treatments. It can lead to reduced quality of life, treatment non-compliance, and nutritional deficiencies. Effective management of nausea is essential for improving patient comfort, maintaining nutritional intake, and ensuring that patients can continue with their prescribed treatment regimens.
- **Opioids**: Opioids are frequently used in cancer care for the management of severe pain, particularly in advanced stages of the disease. While effective in pain control, opioid use is associated with risks such as addiction, tolerance, and adverse side effects. Understanding the role of opioids in cancer treatment, and their interaction with other therapies like cannabis, is crucial for optimizing pain management while minimizing potential harm.
- **Pain**: Pain management is a critical component of cancer treatment, as pain can significantly reduce quality of life and hinder treatment adherence. Effective pain control is essential for allowing patients to maintain daily activities and continue their treatment regimen. Research into various pain management strategies, including the use of cannabis, is important for developing comprehensive care plans that address both the physical and emotional aspects of cancer.
- **Immune Therapy**: Immune therapy, or immunotherapy, represents a significant advancement in cancer treatment, utilizing the body's immune system to target and destroy cancer cells. It is particularly relevant in the treatment of cancers that are resistant to traditional therapies. Understanding the interactions between immune therapy and other treatments, such as cannabis, is critical for optimizing patient outcomes and potentially enhancing the efficacy of immunotherapy.

Cancer Dynamics

- **Anti-Carcinogenic**: Anti-carcinogenic properties refer to the ability of a substance to prevent, inhibit, or reverse the development of cancer. Research into anti-carcinogenic effects is vital for identifying potential preventive strategies and therapeutic agents that can

reduce cancer risk or slow disease progression. In the context of cannabis, exploring anti-carcinogenic effects could reveal novel approaches to cancer prevention and treatment.

- **Cancerous**: The term "cancerous" generally refers to the nature and behavior of cancer cells, including their growth, invasion, and spread to other parts of the body. Understanding the characteristics of cancerous cells is fundamental to developing targeted therapies that can effectively halt or reverse tumor progression. Research in this area focuses on identifying key pathways and mechanisms that drive malignancy.
- **Cancers**: The study of cancers encompasses the exploration of various types of cancer, their etiology, progression, and response to treatment. This broad category is central to cancer research, as it includes investigations into the unique characteristics of different cancer types, which informs the development of specific diagnostic tools and therapeutic strategies.
- **Tumor Growth**: Tumor growth is a key indicator of cancer progression and a primary target for therapeutic intervention. Controlling or inhibiting tumor growth is a major goal of cancer treatment, and research in this area focuses on understanding the factors that promote or inhibit this process. Effective management of tumor growth can lead to improved patient outcomes and longer survival.
- **Tumor Size**: Tumor size is an important metric in cancer diagnosis, staging, and treatment planning. It is often used as a measure of treatment efficacy, with reductions in tumor size indicating a positive response to therapy. Research into factors that influence tumor size, including therapeutic interventions like cannabis, is essential for optimizing treatment strategies.
- **Remission**: Remission refers to the reduction or disappearance of signs and symptoms of cancer, either partially or completely. Achieving remission is the primary goal of cancer treatment, as it is associated with improved survival and quality of life. Research into factors that contribute to remission, including the potential role of cannabis, is crucial for developing effective cancer therapies that offer long-term control or cure of the disease.

Results

Keywords	Supported Occ	SO p	Not supported Occ	NO p	Unclear Occ	UO p	Supported Dom	SD p	Not supported Dom	ND p	Unclear Dom	UD p
**Unclear Dom**	-0.123288782	2.568E-37	-0.087422257	1.651E-19	0.369684039	0						
**Supported Dom**	0.585866514	0	-0.274465797	3.25E-183	-0.071240068	1.877E-13						
**Pain**	0.155864995	7.527E-59	0.049962846	2.516E-07	0.065035409	1.88E-11	0.069339676	8.037E-13	-0.033939665	0.0004624	0.001809349	0.851957
**Opioids**	0.056888388	4.295E-09	0.015289735	0.1147672	0.062561131	1.053E-10	-0.011567692	0.2328037	-0.009278035	0.3385744	0.017001236	0.0794842
**Remission**	0.014287489	0.140554	0.036993025	0.0001351	0.053688132	2.998E-08	-0.002709726	0.7798688	-0.006065576	0.5315589	-0.006505577	0.5022126

**Opioid addiction**	0.054398577	1.966E-08	0.022964811	0.0178375	0.053540047	3.271E-08	-0.004676396	0.629564	-0.001088686	0.9105931	0.010169116	0.2942235
**Therapeutic**	0.481266879	0	0.065204527	1.667E-11	0.044790064	3.799E-06	0.248491385	1.76E-149	-0.116133665	2.821E-33	-0.047471976	9.624E-07
**Addiction**	0.051761063	9.174E-08	0.036640931	0.0001565	0.042410341	1.207E-05	-0.023092973	0.0172096	0.018661348	0.0542347	0.00241934	0.8029442
**Cannabis Types**	0.202338148	1.019E-98	0.103188474	1.381E-26	0.034641323	0.0003515	0.097635382	5.84E-24	0.000146526	0.9879419	-0.024381698	0.0118972
**Anti carcino**	0.088297418	7.167E-20	0.010094706	0.2977708	-0.021848555	0.024209	0.057674671	2.621E-09	-0.014378044	0.1380551	-0.009988428	0.3028865
**Cancers**	0.101808212	6.406E-26	0.017056704	0.078508	-0.019604744	0.0431466	0.080106767	1.276E-16	-0.033432438	0.0005621	-0.018810831	0.0523336
**Quality of life**	0.108646114	2.612E-29	0.020947581	0.030708	0.01916021	0.0481067	0.070186358	4.224E-13	-0.029972783	0.0019869	-0.019145915	0.0482738
**Not supported Dom**	-0.282038394	8.86E-194	0.562419716	0	-0.017959058	0.0639537						
**Tumor growth**	0.037471408	0.0001105	-0.010376168	0.2845028	-0.017060618	0.0784395	0.040946263	2.388E-05	-0.0129997	0.1799575	-0.008976438	0.3545102
**Anti inflammatory**	0.077369933	1.326E-15	0.003316159	0.7323209	-0.015853129	0.101998	0.053566541	3.221E-08	-0.01604299	0.0979589	-0.018943997	0.0506873
**Cancerous**	0.029745913	0.0021495	0.0166817	0.0853013	-0.01488265	0.1247524	0.014719751	0.128933	0.015544551	0.1088441	-0.012494464	0.1974787
**Survival**	0.009269096	0.33904	-0.012715371	0.18967	0.012979392	0.1806386	0.016438927	0.0899472	-0.012553669	0.1953632	0.010335158	0.2864107
**Anxiety**	0.039095742	5.484E-05	0.006475943	0.5041618	0.010906421	0.2606079	0.018328485	0.0586757	0.007431456	0.4433705	-0.002901474	0.7647358
**Chemotherapy**	0.087652678	1.326E-19	0.052815677	4.998E-08	-0.008924829	0.3572839	0.046436636	1.65E-06	0.001876738	0.8465105	-0.02304982	0.0174188
**Nausea**	0.079137982	2.95E-16	0.046552667	1.554E-06	0.008632121	0.3732724	0.037099834	0.0001292	0.002973138	0.7591027	-0.016517683	0.0884182
**Appetite**	0.066645646	5.915E-12	0.034274605	0.0004059	0.007394734	0.4456267	0.032947668	0.0006757	0.006983967	0.4713053	-0.008400073	0.3862572
**Cytokines**	-0.002687101	0.78166	-0.003316856	0.7322668	0.006956788	0.4730327	-0.000598853	0.9507479	-0.015878003	0.1014614	0.002066894	0.8311818
**Depression**	0.008628122	0.3734939	0.007582227	0.434176	0.006183125	0.5236346	0.007788492	0.4217774	0.007886576	0.4159552	0.005768634	0.5518437
**Tumor shrink**	-0.008611567	0.3744115	-0.017926483	0.0644374	-0.005406366	0.5770947	0.008233871	0.3957247	-0.015153337	0.1180404	0.012964065	0.1811541
**Immune therapy**	0.013710506	0.1573001	0.006742461	0.4867757	0.004471678	0.6446372	0.021959616	0.023497	-0.00313693	0.7462761	-0.007383186	0.4463375
**Endocannabinoids**	0.127126718	1.383E-39	-0.001002737	0.9176253	-0.003062692	0.7520812	0.08881736	4.349E-20	-0.038233674	7.981E-05	-0.016926777	0.08081
**Tumor size**	0.021666888	0.0254139	0.003232547	0.7388204	-0.002520875	0.7948551	0.006635688	0.4937018	-0.004874473	0.6151251	-0.005892123	0.5433619
**Radiation therapy**	0.026018291	0.0072734	0.007778554	0.42237	-7.2957E-05	0.993996	0.015739891	0.1044694	-0.003518914	0.7166401	-0.008282236	0.3929553
**Inflammatory**	0.061228658	2.595E-10	-0.009391897	0.3326796	-5.00599E-05	0.9958803	0.051397408	1.128E-07	-0.028086494	0.0037615	-0.01446662	0.135644
**Unclear Occ**							-0.071240068	1.877E-13	-0.017959058	0.0639537	0.369684039	0
**Supported Occ**							0.585866514	0	-0.282038394	8.86E-194	-0.123288782	2.568E-37
**Not supported Occ**							-0.274465797	3.25E-183	0.562419716	0	-0.087422257	1.651E-19

Table 9 Complete table of all topic correlation strengths and p-values. "Occ" = Keyword Occurrences; "Dom" = Dominant Instances; "SO p" = Supported Occurrences p-value; "NO p" = Not supported Occurrences p-value; "UO p" = Unclear Occurrences p-value; "SD p" = Supported Dominant p-value; “ND p” = Not supported Dominant p-value; “UD p” = Unclear dominant p-value

Keyword Occurrence

Topics	Supported KO	SO p	Not Supported KO	NO p	Unclear KO	UO p
**Pain**	0.155864995	7.527E-59	0.049962846	2.516E-07	0.065035409	1.88E-11
**Opioids**	0.056888388	4.295E-09	0.015289735	0.1147672	0.062561131	1.053E-10
**Remission**	0.014287489	0.140554	0.036993025	0.0001351	0.053688132	2.998E-08
**Opioid addiction**	0.054398577	1.966E-08	0.022964811	0.0178375	0.053540047	3.271E-08
**Therapeutic**	0.481266879	0	0.065204527	1.667E-11	0.044790064	3.799E-06
**Addiction**	0.051761063	9.174E-08	0.036640931	0.0001565	0.042410341	1.207E-05
**Cannabis Types**	0.202338148	1.019E-98	0.103188474	1.381E-26	0.034641323	0.0003515
**Anti carcino**	0.088297418	7.167E-20	0.010094706	0.2977708	-0.021848555	0.024209
**Cancers**	0.101808212	6.406E-26	0.017056704	0.078508	-0.019604744	0.0431466
**Quality of life**	0.108646114	2.612E-29	0.020947581	0.030708	0.01916021	0.0481067
**Negative Dom**	-0.282038394	8.86E-194	0.562419716	0	-0.017959058	0.0639537
**Tumor growth**	0.037471408	0.0001105	-0.010376168	0.2845028	-0.017060618	0.0784395
**Anti inflammatory**	0.077369933	1.326E-15	0.003316159	0.7323209	-0.015853129	0.101998
**Cancerous**	0.029745913	0.0021495	0.0166817	0.0853013	-0.01488265	0.1247524
**Survival**	0.009269096	0.33904	-0.012715371	0.18967	0.012979392	0.1806386
**Anxiety**	0.039095742	5.484E-05	0.006475943	0.5041618	0.010906421	0.2606079
**Chemotherapy**	0.087652678	1.326E-19	0.052815677	4.998E-08	-0.008924829	0.3572839
**Nausea**	0.079137982	2.95E-16	0.046552667	1.554E-06	0.008632121	0.3732724
**Appetite**	0.066645646	5.915E-12	0.034274605	0.0004059	0.007394734	0.4456267
**Cytokines**	-0.002687101	0.78166	-0.003316856	0.7322668	0.006956788	0.4730327
**Depression**	0.008628122	0.3734939	0.007582227	0.434176	0.006183125	0.5236346
**Tumor shrink**	-0.008611567	0.3744115	-0.017926483	0.0644374	-0.005406366	0.5770947
**Immune therapy**	0.013710506	0.1573001	0.006742461	0.4867757	0.004471678	0.6446372
**Endocannabinoids**	0.127126718	1.383E-39	-0.001002737	0.9176253	-0.003062692	0.7520812

**Tumor size**	0.021666888	0.0254139	0.003232547	0.7388204	-0.002520875	0.7948551
**Radiation therapy**	0.026018291	0.0072734	0.007778554	0.42237	-7.2957E-05	0.993996
**Inflammatory**	0.061228658	2.595E-10	-0.009391897	0.3326796	-5.00599E-05	0.9958803

Table 10 List of all topic correlations with sentiments and p-values by dominant instances

Dominant Instances

**Topics**	**Supported DI**	**SPD p**	**Not Supported DI**	**ND p**	**Unclear DI**	**UD p**
**Pain**	0.069339676	8.037E-13	-0.033939665	0.0004624	0.001809349	0.851957
**Opioids**	-0.011567692	0.2328037	-0.009278035	0.3385744	0.017001236	0.0794842
**Remission**	-0.002709726	0.7798688	-0.006065576	0.5315589	-0.006505577	0.5022126
**Opioid addiction**	-0.004676396	0.629564	-0.001088686	0.9105931	0.010169116	0.2942235
**Therapeutic**	0.248491385	1.76E-149	-0.116133665	2.821E-33	-0.047471976	9.624E-07
**Addiction**	-0.023092973	0.0172096	0.018661348	0.0542347	0.00241934	0.8029442
**Cannabis Types**	0.097635382	5.84E-24	0.000146526	0.9879419	-0.024381698	0.0118972
**Anti carcino**	0.057674671	2.621E-09	-0.014378044	0.1380551	-0.009988428	0.3028865
**Cancers**	0.080106767	1.276E-16	-0.033432438	0.0005621	-0.018810831	0.0523336
**Quality of life**	0.070186358	4.224E-13	-0.029972783	0.0019869	-0.019145915	0.0482738
**Negative Dom**						
**Tumor growth**	0.040946263	2.388E-05	-0.0129997	0.1799575	-0.008976438	0.3545102
**Anti inflammatory**	0.053566541	3.221E-08	-0.01604299	0.0979589	-0.018943997	0.0506873
**Cancerous**	0.014719751	0.128933	0.015544551	0.1088441	-0.012494464	0.1974787
**Survival**	0.016438927	0.0899472	-0.012553669	0.1953632	0.010335158	0.2864107
**Anxiety**	0.018328485	0.0586757	0.007431456	0.4433705	-0.002901474	0.7647358
**Chemotherapy**	0.046436636	1.65E-06	0.001876738	0.8465105	-0.02304982	0.0174188
**Nausea**	0.037099834	0.0001292	0.002973138	0.7591027	-0.016517683	0.0884182
**Appetite**	0.032947668	0.0006757	0.006983967	0.4713053	-0.008400073	0.3862572
**Cytokines**	-0.000598853	0.9507479	-0.015878003	0.1014614	0.002066894	0.8311818
**Depression**	0.007788492	0.4217774	0.007886576	0.4159552	0.005768634	0.5518437

**Tumor shrink**	0.008233871	0.3957247	-0.015153337	0.1180404	0.012964065	0.1811541
**Immune therapy**	0.021959616	0.023497	-0.00313693	0.7462761	-0.007383186	0.4463375
**Endocannabinoids**	0.08881736	4.349E-20	-0.038233674	7.981E-05	-0.016926777	0.08081
**Tumor size**	0.006635688	0.4937018	-0.004874473	0.6151251	-0.005892123	0.5433619
**Radiation therapy**	0.015739891	0.1044694	-0.003518914	0.7166401	-0.008282236	0.3929553
**Inflammatory**	0.051397408	1.128E-07	-0.028086494	0.0037615	-0.01446662	0.135644

Table 11 List of all topic correlations with sentiments and p-values by dominant instances

Refined Dataset

**Topics KO**	**Supported**	**SO p**	**Not supported**	**NO p**	**Unclear**	**UO p**
Anti carcinogenic	0.088297	7.17E-20			-0.02185	0.024209
Anti inflammatory	0.07737	1.33E-15				
Appetite	0.066646	5.91E-12	0.034275	0.000406		
Cancerous	0.029746	0.002149				
Cancers	0.101808	6.41E-26			-0.0196	0.043147
Chemotherapy	0.087653	1.33E-19	0.052816	5E-08		
Inflammatory	0.061229	2.59E-10				
Nausea	0.079138	2.95E-16	0.046553	1.55E-06		
Opioids	0.056888	4.3E-09			0.062561	1.05E-10
Pain	0.155865	7.53E-59	0.049963	2.52E-07	0.065035	1.88E-11
Radiation therapy	0.026018	0.007273				
Therapeutic	0.481267	0	0.065205	1.67E-11	0.04479	3.8E-06
Tumor growth	0.037471	0.000111				
Tumor size	0.021667	0.025414				
Remission			0.036993	0.000135	0.053688	3E-08

Table 12 List of topic correlations and p-values by keyword occurrence, filtered for significance and minimum Pearson's r strength

Topics DI	Supported Dom	SD p	Not supported Dom	ND p	Unclear Dom2	UD p
Anti carcinogenic	0.057675	2.62E-09				
Anti inflammatory	0.053567	3.22E-08				
Appetite	0.032948	0.000676				
Cancers	0.080107	1.28E-16	-0.03343	0.000562		
Chemotherapy	0.046437	1.65E-06			-0.02305	0.017419
Immune therapy	0.02196	0.023497				
Inflammatory	0.051397	1.13E-07	-0.02809	0.003762		
Nausea	0.0371	0.000129				
Pain	0.06934	8.04E-13	-0.03394	0.000462		
Therapeutic	0.248491	1.8E-149	-0.11613	2.82E-33	-0.04747	9.62E-07
Tumor growth	0.040946	2.39E-05				

Table 13 List of topic correlations and p-values by dominant instances, filtered for significance and minimum Pearson's r strength

Cannabis Types

Topics	Supported	SO p	Not supported	NO p	Unclear	UO p
Cannabis Types	0.202338	1.02E-98	0.103188	1.38E-26	0.034641	0.000351
Endocannabinoids	0.127127	1.38E-39				

Table 14 List of cannabinoid types topic correlations and p-values by keyword occurrences, filtered for significance and minimum Pearson's r strength

Cannabinoids Keywords	Supported KO	p KO
Cannabis Types Sum	0.202338	1.01934E-98
cannabinoids	0.199254	9.92388E-96
cbd	0.143145	7.96879E-50

cannabidiol	0.132784	4.6395E-43
cannabis sativa	0.109566	8.79381E-30
phytocannabinoids	0.08469	2.11711E-18
endocannabinoids	0.075578	5.87909E-15
tetrahydrocannabinol	0.060668	3.77067E-10
cannabinol	0.055496	1.01346E-08
cbg	0.04405	5.47616E-06
cbc	0.042329	1.25466E-05
cannabis ruderalis	0.034443	0.000380025
cannabichromene	0.03271	0.000739041
cannabis indica	0.032496	0.000800623
thc	0.028485	0.003296582
cannabis concentrate	-0.01985	0.04058015

Table 15 list of cannabinoid individual keywords grouped by Supported sentiment by keyword occurrence

Cannabinoids Keywords	Not Supported KO	p KO
Cannabis Types Sum	0.103188	1.38112E-26
cannabinoids	0.083572	5.87182E-18
thc	0.080828	6.79389E-17
cannabidiol	0.072582	6.56909E-14
tetrahydrocannabinol	0.071839	1.1777E-13
cannabinol	0.069077	9.79335E-13
cannabis sativa	0.049403	3.41979E-07
cannabichromene	0.039768	4.07167E-05
cbd	0.039462	4.66531E-05
phytocannabinoids	0.032506	0.000797658
delta-9-tetrahydrocannabinol	0.03169	0.001077513

Table 16 list of cannabinoid individual keywords grouped by Not Supported sentiment by keyword occurrence

Cannabinoids Keywords	Unclear KO	p KO
cannabinoids	0.03654	0.000163167
Cannabis Types Sum	0.034641	0.000351453
cannabidiol	0.025576	0.008328434
cbd	0.02273	0.019040954
tetrahydrocannabivarin	-0.01918	0.047891222

Table 17 list of cannabinoid individual keywords grouped by Unclear sentiment by keyword occurrence

Health Metrics Results

Focusing on the role of inflammation and therapeutic interventions in cancer progression and treatment outcomes, with an emphasis on how these factors influence patient survival and quality of life. ***Table 8*** cross references the topic correlations with the sentiment analyses by keyword occurrence, while ***Table 9*** cross references topic correlations with the sentiment analyses by dominant instances. ***Table 10*** provides further information on the individual keywords that constitute the different topics.

**Health Metrics Topics KO**	**Supported**	**SO p**	**Not supported**	**NO p**	**Unclear**	**UO p**
Anti inflammatory	0.07737	1.33E-15				
Inflammatory	0.061229	2.59E-10				
Therapeutic	0.481267	0	0.065205	1.67E-11	0.04479	3.8E-06

Table 18 Topic correlations with the sentiment analyses by keyword occurrence (KO). SO p: Supported by keyword occurrence p-value, NO p: Not supported by keyword occurrence p-value, UO p: Unclear by keyword occurrence p-value. Black cells did not qualify by either minimum r or p values.

**Health Metrics Topics DI**	**Supported Dom**	**SD p**	**Not supported Dom**	**ND p**	**Unclear Dom2**	**UD p**
Anti inflammatory	0.053567	3.22E-08				
Inflammatory	0.051397	1.13E-07	-0.02809	0.003762		
Therapeutic	0.248491	1.8E-149	-0.11613	2.82E-33	-0.04747	9.62E-07

Table 19 Topic correlations with the sentiment analyses by dominant instances (DI). SD p: Supported by dominant instances p-value, ND p: Not supported by dominant instances p-value, UD p: Unclear by dominant instances p-value. Black cells did not qualify by either minimum r or p values.

**Health Metrics KW**	**Supported r**	**S p**	**Not Supported r**	**N p**	**Unclear r**	**U p**

Anti-inflammatory	0.075492	6.31E-15				
Chronic inflammatory disease	0.019805	0.041055				
Chronic inflammatory process	0.020071	0.038415	0.0219967	0.023264		
C-X-C motif chemokine ligand	0.034443	0.00038				
Cytokine	0.042436	1.19E-05				
Cytokines	0.038142	8.3E-05				
EPO	-0.02537	0.008873				
IL-6	0.02191	0.023814				
Immunomodulatory	0.036514	0.000165				
Inflammation	0.063872	4.26E-11				
Inflammatory process	0.027094	0.005189				
Inflammatory response	0.022057	0.022887				
Pro-inflammatory	0.021522	0.02641				
TNF-alpha	0.019309	0.046393				

Table 20 Individual keyword (KW) correlations with the sentiment analyses by keyword occurrence. r: Pearson's correlation coefficient (association strength), S p: Supported by keyword occurrence p-value, N p: Not supported by keyword occurrence p-value, U p: Unclear by keyword occurrence p-value. Black cells did not qualify by either minimum r or p values.

Cancer Treatments Results

Examining the effectiveness of cannabis in managing cancer-related symptoms like appetite loss, pain, and nausea, as well as its interaction with standard treatments such as chemotherapy and immunotherapy. ***Table 11*** cross references the topic correlations with the sentiment analyses by keyword occurrence, while ***Table 12*** cross references topic correlations with the sentiment analyses by dominant instances. ***Table 13*** provides further information on the individual keywords that constitute the different topics.

**Treatment Topics KO**	**Supported**	**SO p**	**Not supported**	**NO p**	**Unclear**	**UO p**
Appetite	0.066646	5.91E-12	0.034275	0.000406		
Chemotherapy	0.087653	1.33E-19	0.052816	5E-08		
Nausea	0.079138	2.95E-16	0.046553	1.55E-06		
Opioids	0.056888	4.3E-09			0.062561	1.05E-10
Pain	0.155865	7.53E-59	0.049963	2.52E-07	0.065035	1.88E-11

Table 21 Topic correlations with the sentiment analyses by keyword occurrence (KO). SO p: Supported by keyword occurrence p-value, NO p: Not supported by keyword occurrence p-value, UO p: Unclear by keyword occurrence p-value. Black cells did not qualify by either minimum r or p values.

Treatment Topics DI	Supported Dom	SD p	Not supported Dom	ND p	Unclear Dom2	UD p
Appetite	0.032948	0.000676				
Chemotherapy	0.046437	1.65E-06			-0.02305	0.017419
Immune therapy	0.02196	0.023497				
Nausea	0.0371	0.000129				
Pain	0.06934	8.04E-13	-0.03394	0.000462		

Table 22 Topic correlations with the sentiment analyses by dominant instances (DI). SD p: Supported by dominant instances p-value, ND p: Not supported by dominant instances p-value, UD p: Unclear by dominant instances p-value. Black cells did not qualify by either minimum r or p values.

Cancer Treatments KW	Supported r	S p	Not Supported r	N p	Unclear r	U p
chemo	0.092438	1.24E-21	0.0482069	6.52E-07		
Chemopreventive	0.038773	6.32E-05				
Chemotherapeutic	0.099062	1.28E-24	0.0362626	0.000183		
chemotherapeutic treatment	0.028386	0.003407				
chemotherapy	0.069225	8.76E-13	0.0513557	1.15E-07		
Radiotherapy	0.022783	0.018762				

Table 23 Individual keyword (KW) correlations with the sentiment analyses by keyword occurrence. r: Pearson's correlation coefficient (association strength), S p: Supported by keyword occurrence p-value, N p: Not supported by keyword occurrence p-value, U p: Unclear by keyword occurrence p-value. Black cells did not qualify by either minimum r or p values.

Cancer Dynamics Results

Investigating the impact of cannabis on cancer progression, including anti-carcinogenic effects, tumor growth, size, and remission rates, to understand its potential as a complementary treatment in oncology. ***Table 14*** cross references the topic correlations with the sentiment analyses by keyword occurrence, while ***Table 15*** cross references topic correlations with the sentiment analyses by dominant instances. ***Table 16*** provides further information on the individual keywords that constitute the different topics.

Cancer Dynamics Topics KO	Supported	SO p	Not supported	NO p	Unclear	UO p

Anti carcinogenic	0.088297	7.17E-20			-0.02185	0.024209
Cancerous	0.029746	0.002149				
Cancers	0.101808	6.41E-26			-0.0196	0.043147
Tumor growth	0.037471	0.000111				
Tumor size	0.021667	0.025414				
Remission			0.036993	0.000135	0.053688	3E-08

Table 24 Topic correlations with the sentiment analyses by keyword occurrence (KO). SO p: Supported by keyword occurrence p-value, NO p: Not supported by keyword occurrence p-value, UO p: Unclear by keyword occurrence p-value. Black cells did not qualify by either minimum r or p values.

**Cancer Dynamics Topics DI**	**Supported Dom**	**SD p**	**Not supported Dom**	**ND p**	**Unclear Dom2**	**UD p**
Anti carcinogenic	0.057675	2.62E-09				
Cancers	0.080107	1.28E-16	-0.03343	0.000562		
Tumor growth	0.040946	2.39E-05				

Table 25 Topic correlations with the sentiment analyses by dominant instances (DI). SD p: Supported by dominant instances p-value, ND p: Not supported by dominant instances p-value, UD p: Unclear by dominant instances p-value. Black cells did not qualify by either minimum r or p values.

**Cancer Dynamics KW**	**Supported r**	**S p**	**Not Supported r**	**N p**	**Unclear r**	**U p**
Anticarcinogenic	0.026291	0.006683				
Antineoplastic	0.020933	0.030824				
Antitumor	0.055215	1.2E-08				
Antitumoral	0.027711	0.004253				
Cancer	0.100197	3.74E-25	0.0277028	0.004264		
Cancers	0.035054	0.000298	0.0285136	0.003265		
Carcinogenesis	0.019705	0.042086				
Carcinogenic			0.0237177	0.014419		
Clinical remission			0.0498664	2.65E-07	0.067801	2.54E-12
Glioma	0.023074	0.017303				
Malignancy	0.02142	0.027137				
Metastasis					-0.02124	0.028464
Neoplasm	0.026601	0.006066				

Neoplastic	0.034986	0.000307			-0.0199	0.040051
Oncology	0.049223	3.77E-07				
Remission			0.0311017	0.001333	0.046295	1.77E-06
Tumor	0.053518	3.31E-08			-0.02171	0.025153

Table 26 Individual keyword (KW) correlations with the sentiment analyses by keyword occurrence. r: Pearson's correlation coefficient (association strength), S p: Supported by keyword occurrence p-value, N p: Not supported by keyword occurrence p-value, U p: Unclear by keyword occurrence p-value. Black cells did not qualify by either minimum r or p values.

In addition to the correlations of the preceding topics of health metrics, cancer treatments, and cancer dynamics, a refined dataset was created to explore the keywords associated with specific types of cannabis studied across various research articles, as seen in ***Table 17***. This information was deemed relevant to identify any confounders based on endocannabinoids or disrupting outliers, none of which were identified. This supplementary data is available for researchers interested in delving deeper into the specific impacts and characteristics of different cannabis strains and their relevance to the broader findings of this study.

KW KO	Supported r	S p	Not Supported r	N p	Unclear r	U p
cannabichromene	0.03271	0.000739	0.0397676	4.07E-05		
cannabidiol	0.132784	4.64E-43	0.0725819	6.57E-14	0.025576	0.008328
cannabinoid-based therapy	0.020384	0.035492				
cannabinoids	0.199254	9.92E-96	0.0835723	5.87E-18	0.03654	0.000163
cannabinol	0.055496	1.01E-08	0.0690774	9.79E-13		
cannabis concentrate	-0.01985	0.04058				
cannabis indica	0.032496	0.000801				
cannabis ruderalis	0.034443	0.00038				
cannabis sativa	0.109566	8.79E-30	0.0494032	3.42E-07		
Cannabis Types Sum	0.202338	1.02E-98	0.1031885	1.38E-26	0.034641	0.000351
cannabis-based medicines	0.029082	0.002698	0.0216603	0.025458	0.070607	3.06E-13
cbc	0.042329	1.25E-05				
cbd	0.143145	7.97E-50	0.0394617	4.67E-05	0.02273	0.019041
cbg	0.04405	5.48E-06				
endocannabinoids	0.075578	5.88E-15				
medicinal cannabis	0.24221	6.8E-142				
phytocannabinoids	0.08469	2.12E-18	0.0325055	0.000798		

tetrahydrocannabinol	0.060668	3.77E-10	0.0718388	1.18E-13		
thc	0.028485	0.003297	0.0808284	6.79E-17		
therapeutic cannabis	0.049169	3.89E-07				

Table 27 Individual keyword correlations with the sentiment analyses by keyword occurrence. r: Pearson's correlation coefficient (association strength), KW KO: Keywords Keyword Occurrence calculation. Black cells did not qualify by either minimum r or p values.

[i] De Feo G, Case AA, Crawford GB, et al. Multinational Association of Supportive Care in Cancer (MASCC) guidelines: cannabis for psychological symptoms including insomnia, anxiety, and depression. *Support Care Cancer*. 2023;31(3):176. Published 2023 Feb 21. doi:10.1007/s00520-023-07628-3

[ii] To J, Davis M, Sbrana A, et al. MASCC guideline: cannabis for cancer-related pain and risk of harms and adverse events [published correction appears in Support Care Cancer. 2023 May 6;31(6):323. doi: 10.1007/s00520-023-07789-1]. *Support Care Cancer*. 2023;31(4):202. Published 2023 Mar 6. doi:10.1007/s00520-023-07662-1

[iii] Zeraatkar D, Cooper MA, Agarwal A, et al. Long-term and serious harms of medical cannabis and cannabinoids for chronic pain: a systematic review of non-randomised studies. *BMJ Open*. 2022;12(8):e054282. Published 2022 Aug 4. doi:10.1136/bmjopen-2021-054282

[iv] Lee C, Danielson EC, Beestrum M, Eurich DT, Knapp A, Jordan N. Medical Cannabis and Its Efficacy/Effectiveness for the Treatment of Low-Back Pain: a Systematic Review. *Curr Pain Headache Rep*. 2023;27(12):821-835. doi:10.1007/s11916-023-01189-0

[v] Hanganu B, Lazar DE, Manoilescu IS, et al. Controversial Link between Cannabis and Anticancer Treatments-Where Are We and Where Are We Going? A Systematic Review of the Literature. *Cancers (Basel)*. 2022;14(16):4057. Published 2022 Aug 22. doi:10.3390/cancers14164057

[vi] Valenti C, Billi M, Pancrazi GL, et al. Biological effects of cannabidiol on human cancer cells: Systematic review of the literature. *Pharmacol Res*. 2022;181:106267. doi:10.1016/j.phrs.2022.106267

[vii] Bachari A, Piva TJ, Salami SA, Jamshidi N, Mantri N. Roles of Cannabinoids in Melanoma: Evidence from In Vivo Studies. *Int J Mol Sci*. 2020;21(17):6040. Published 2020 Aug 21. doi:10.3390/ijms21176040

[viii] De Feo G, Case AA, Crawford GB, et al. Multinational Association of Supportive Care in Cancer (MASCC) guidelines: cannabis for psychological symptoms including insomnia, anxiety, and depression. *Support Care Cancer*. 2023;31(3):176. Published 2023 Feb 21. doi:10.1007/s00520-023-07628-3

[ix] To J, Davis M, Sbrana A, et al. MASCC guideline: cannabis for cancer-related pain and risk of harms and adverse events [published correction appears in Support Care Cancer. 2023 May 6;31(6):323. doi: 10.1007/s00520-023-07789-1]. *Support Care Cancer*. 2023;31(4):202. Published 2023 Mar 6. doi:10.1007/s00520-023-07662-1

[x] Zeraatkar D, Cooper MA, Agarwal A, et al. Long-term and serious harms of medical cannabis and cannabinoids for chronic pain: a systematic review of non-randomised studies. *BMJ Open*. 2022;12(8):e054282. Published 2022 Aug 4. doi:10.1136/bmjopen-2021-054282

[xi] Lee C, Danielson EC, Beestrum M, Eurich DT, Knapp A, Jordan N. Medical Cannabis and Its Efficacy/Effectiveness for the Treatment of Low-Back Pain: a Systematic Review. *Curr Pain Headache Rep*. 2023;27(12):821-835. doi:10.1007/s11916-023-01189-0

[xii] Hanganu B, Lazar DE, Manoilescu IS, et al. Controversial Link between Cannabis and Anticancer Treatments-Where Are We and Where Are We Going? A Systematic Review of the Literature. *Cancers (Basel)*. 2022;14(16):4057. Published 2022 Aug 22. doi:10.3390/cancers14164057

[xiii] Valenti C, Billi M, Pancrazi GL, et al. Biological effects of cannabidiol on human cancer cells: Systematic review of the literature. *Pharmacol Res*. 2022;181:106267. doi:10.1016/j.phrs.2022.106267

[xiv] Bachari A, Piva TJ, Salami SA, Jamshidi N, Mantri N. Roles of Cannabinoids in Melanoma: Evidence from In Vivo Studies. *Int J Mol Sci*. 2020;21(17):6040. Published 2020 Aug 21. doi:10.3390/ijms21176040

[xv] Mead, A. (2017). The legal status of cannabis (marijuana) and cannabidiol (CBD) under US law. *Epilepsy & Behavior*, *70*, 288-291.

[xvi] Maayah, Z. H., Takahara, S., Ferdaoussi, M., & Dyck, J. R. (2020). The anti-inflammatory and analgesic effects of formulated full-spectrum cannabis extract in the treatment of neuropathic pain associated with multiple sclerosis. *Inflammation Research*, *69*, 549-558.

[xvii] Pennypacker, S. D., & Romero-Sandoval, E. A. (2020). CBD and THC: do they complement each other like Yin and Yang?. *Pharmacotherapy: The Journal of Human Pharmacology and Drug Therapy*, *40*(11), 1152-1165.

[xviii] Freeman, A. M., Petrilli, K., Lees, R., Hindocha, C., Mokrysz, C., Curran, H. V., ... & Freeman, T. P. (2019). How does cannabidiol (CBD) influence the acute effects of delta-9-tetrahydrocannabinol (THC) in humans? A systematic review. *Neuroscience & Biobehavioral Reviews*, *107*, 696-712.

[xix] Baron, E. P. (2018). Medicinal properties of cannabinoids, terpenes, and flavonoids in cannabis, and benefits in migraine, headache, and pain: an update on current evidence and cannabis science. *Headache: The Journal of Head and Face Pain*, *58*(7), 1139-1186.

[xx] Zhao, Y. J., Cheng, Q. Q., Su, P., Chen, X., Wang, X. J., Gao, W., & Huang, L. Q. (2014). Research progress relating to the role of cytochrome P450 in the biosynthesis of terpenoids in medicinal plants. *Applied microbiology and biotechnology*, *98*, 2371-2383.

[xxi] Cohen, J., Cohen, P., West, S.G., & Aiken, L.S. (2003). Applied Multiple Regression/Correlation Analysis for the Behavioral Sciences.

[xxii] Ratner, B. The correlation coefficient: Its values range between +1/−1, or do they?. J Target Meas Anal Mark 17, 139–142 (2009). https://doi.org/10.1057/jt.2009.5

[xxiii] Rodgers, J. L., & Nicewander, W. A. (1988). Thirteen ways to look at the correlation coefficient. The American Statistician, 42(1), 59-66.
